# The Unfolded Protein Responses in Health, Aging, and Neurodegeneration: Recent Advances and Future Considerations

**DOI:** 10.3389/fnmol.2022.831116

**Published:** 2022-02-25

**Authors:** Andrew P. K. Wodrich, Andrew W. Scott, Arvind Kumar Shukla, Brent T. Harris, Edward Giniger

**Affiliations:** ^1^National Institute of Neurological Disorders and Stroke, National Institutes of Health, Bethesda, MD, United States; ^2^Interdisciplinary Program in Neuroscience, Georgetown University, Washington, DC, United States; ^3^College of Medicine, University of Kentucky, Lexington, KY, United States; ^4^Department of Pathology, Georgetown University, Washington, DC, United States; ^5^Department of Neurology, Georgetown University, Washington, DC, United States

**Keywords:** unfolded protein response (UPR), mitochondrial unfolded protein response (UPR*^mt^*), endoplasmic reticulum unfolded protein response, aging, neurodegeneration

## Abstract

Aging and age-related neurodegeneration are both associated with the accumulation of unfolded and abnormally folded proteins, highlighting the importance of protein homeostasis (termed proteostasis) in maintaining organismal health. To this end, two cellular compartments with essential protein folding functions, the endoplasmic reticulum (ER) and the mitochondria, are equipped with unique protein stress responses, known as the ER unfolded protein response (UPR*^ER^*) and the mitochondrial UPR (UPR*^mt^*), respectively. These organellar UPRs play roles in shaping the cellular responses to proteostatic stress that occurs in aging and age-related neurodegeneration. The loss of adaptive UPR*^ER^* and UPR*^mt^* signaling potency with age contributes to a feed-forward cycle of increasing protein stress and cellular dysfunction. Likewise, UPR*^ER^* and UPR*^mt^* signaling is often altered in age-related neurodegenerative diseases; however, whether these changes counteract or contribute to the disease pathology appears to be context dependent. Intriguingly, altering organellar UPR signaling in animal models can reduce the pathological consequences of aging and neurodegeneration which has prompted clinical investigations of UPR signaling modulators as therapeutics. Here, we review the physiology of both the UPR*^ER^* and the UPR*^mt^*, discuss how UPR*^ER^* and UPR*^mt^* signaling changes in the context of aging and neurodegeneration, and highlight therapeutic strategies targeting the UPR*^ER^* and UPR*^mt^* that may improve human health.

## Introduction

Maintaining protein homeostasis (hereafter: proteostasis) within cells is essential for cellular health, especially in post-mitotic cells such as neurons that cannot dilute the ill-effects of misfolded, unfolded, or aggregated proteins through cell division. Several cellular compartments are more intimately in contact with proteins through their role as sites of protein synthesis and folding and are, therefore, more prone to encountering abnormal protein species over the lifetime of a cell. Two such compartments, the endoplasmic reticulum (ER) and the mitochondria, must maintain tight regulation of proteostasis on their specialized cellular functions and thus are equipped with dedicated protein stress responses. These organelles harbor molecular machinery to rapidly detect and mitigate proteotoxic stress through conserved signaling pathways that elicit defined transcriptional responses to promote proteostasis and sustain organellar and cellular health. Collectively, these elaborate networks of proteotoxic stress detection, signaling, and alleviation are termed the ER unfolded protein response (UPR*^ER^*) and the mitochondrial UPR (UPR*^mt^*), respectively. An accumulation of UPR research combining *in vitro*, *in vivo*, and human post-mortem data has revealed that these processes are intimately entwined with both aging and neurodegeneration. Here, we will review the core machinery responsible for UPR*^ER^* and UPR*^mt^* function; how these components are affected with age and in age-related neurodegenerative disease (NDD); and how their genetic or pharmacological manipulation can, in turn, influence these biological processes to shape organismal health.

## Physiology of the Unfolded Protein Response

Work primarily done over the past three decades has elucidated how the organellar UPRs, the UPR*^ER^* and the UPR*^mt^*, are activated; how the activation of these UPRs induces targeted cellular responses; and how the UPR-inducing stresses are resolved. Here, we focus on the core biological pathways that define the UPR*^ER^* and UPR*^mt^*, emphasizing the machinery relevant to aging and neurodegeneration. Thus, we will unfortunately omit the work of many, which can be reviewed elsewhere for the UPR*^ER^* (Hetz et al., 2020; Metcalf et al., 2020) and the UPR*^mt^* (Kenny and Germain, 2017; Münch, 2018; Shpilka and Haynes, 2018). Please note that when referencing gene or protein names in a specific organism, we will use the nomenclature relevant to the organism; when referencing a gene or protein generally, we will use the human nomenclature. For a complete list of aging- and NDD-associated genes and their orthologs, please see Table 1.

**TABLE 1:** UPR*^ER^*- and UPR*^mt^*-related genes with relevance in aging or neurodegeneration and their orthologs in humans (*Homo sapiens*), house mice (*Mus musculus*), fruit flies (*Drosophila melanogaster*), nematodes (*Caenorhabditis elegans*), and yeast (*Saccharomyces cerevisiae*).

Functional class	*H. sapiens*	*M. musculus*	*D. melanogaster*	*C. elegans*	*S. cerevisiae*
Chaperone	HSPA5 (BiP)	Hspa5 (BiP)	Hsc70-3 (BiP)	hsp-4 (BiP)	KAR2 (BiP)
	HSPA9 (mtHSP70)	Hspa9 (mtHsp70)	Hsc70-5	hsp-6	SSC1^†^
	HSPD1 (HSP60)	Hspd1 (Hsp60)	Hsp60A	hsp-60	HSP60^†^
	HSPE1 (HSP10)	Hspe1 (Hsp10)	CG9920^†^/CG11267^†^	Y22D7AL.10^†^	HSP10^†^
	P4HB (PDI)	P4hb (PDI)	Pdi	pdi-2	PDI1
	TRAP1	Trap1	Trap1	hsp-75^†^	HSC82^†^
Deacetylase	SIRT3	Sirt3	–	–	–
Epigenetic regulator	BAZ2A/BAZ2B	Baz2a/Baz2b	tou^†^	baz-2	–
	EHMT1/EHMT2	Ehmt1/Ehmt2	–	set-6	–
	HDAC1^†^/HDAC2^†^	Hdac1^†^/Hdac2^†^	HDAC1^†^	hda-1	–
	KDM6B	Kdm6b	Utx	jmjd-3.1	CYC8^†^
	PHF8	Phf8	–	jmjd-1.1/jmjd-1.2	JHD1^†^
	SETDB1	Setdb1	egg^†^	met-2	–
Growth Factor	GDF15	Gdf15	–	–	–
Hormone	FGF21	Fgf21	–	–	–
Hormone receptor	ESR1 (ERα)	Esr1 (ERα)	ERR^†^ (ERα)	–	–
Kinase	EIF2AK3 (PERK)	Eif2ak3 (PERK)	Pek	pek-1	–
Kinase/endoribonuclease	ERN1 (IRE1α)	Ern1 (Ire1α)	Ire1	ire-1	IRE1
Protease	CLPP	Clpp	ClpP	clpp-1	–
	HTRA2	Htra2	HtrA2	–	–
	LONP1	Lonp1	Lon	lonp-1	PIM1^†^
	YMEL1L	Yme1l1	YME1L	ymel-1	YME1^†^
Protein phosphatase subunit	PPP1R15A (GADD34)	Ppp1r15a (Gadd34)	PPP1R15A (GADD34)	–	–
Transcription factor	ATF4	Atf4	–	–	–
	ATF5	Atf5	–	atfs-1	–
	ATF6	Atf6	Atf6	atf-6	–
	CREBBP (CBP)/EP300 (p300)	Crebbp (CBP)/Ep300 (p300)	–	cbp-1	–
	DDIT3 (CHOP)	Ddit3 (CHOP)	–	–	–
	FOXO3	Foxo3	foxo	daf-16	HCM1^†^
	SATB1^†^/SATB2^†^	Satb1^†^/Satb2^†^	dve	dve-1	–
	XBP1	Xbp1	Xbp1	xbp-1	HAC1
Translation initiation factor	EIF2S1 (eIF2α)	Eif2s1 (eIF2α)	eIF2alpha	eif-2alpha	SUI2^†^ (eIF2α)
Ubiquitin-like protein	UBL5^†^	Ubl5^†^	ubl^†^	ubl-5	HUB1^†^

*Gene orthologs have been obtained from the literature (as cited in the text) and ENSEMBL (v104; https://www.ensembl.org) (Howe et al., 2020). In cases where another name or abbreviation is more commonly used in the literature, we have noted the more commonly used name in parentheses and will use this more common terminology throughout the text. The dagger symbol (^†^) indicates genes that are orthologs of confirmed UPR^ER^- or UPR^mt^-related genes that have yet to be validated experimentally in that particular species.*

### Physiology of the Endoplasmic Reticulum Unfolded Protein Response

#### Mechanism of the Endoplasmic Reticulum Unfolded Protein Response

##### Endoplasmic Reticulum Unfolded Protein Response Activation

The first mechanistic step of the UPR*^ER^* is the sensing of ER stress. The protein folding environment within the ER is sensitive to a range of internal and external stressors that can trigger ER stress. Hypoxia, redox or calcium imbalance, nutrient deprivation and viral challenge, as well as errors of translation and expression of genetic mutants, can all disrupt ER homeostasis and lead to the accumulation of misfolded proteins within the ER lumen (Wang and Kaufman, 2016). The convergence of diverse stimuli on proteotoxic stress establishes it as the primary trigger of the UPR*^ER^*, though recent work has also demonstrated that impaired lipid metabolism can activate the UPR*^ER^* in a manner distinct from protein misfolding (Hou et al., 2014).

##### Endoplasmic Reticulum Unfolded Protein Response Effectors and Effects

**FIGURE 1:**
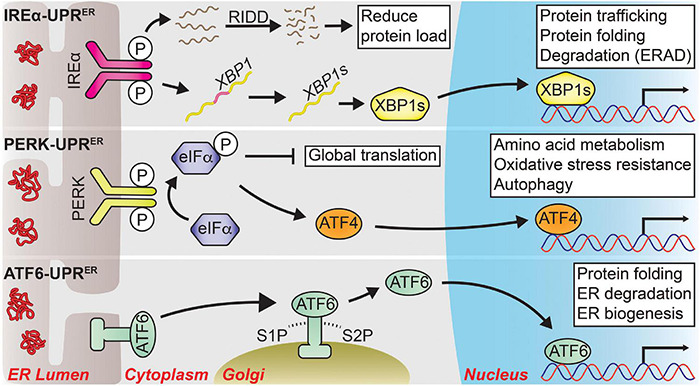
The three major signal transduction pathways of the UPR*^ER^*. Following ER stress, three distinct branches are activated that shape the UPR*^ER^*. *IRE1α-UPR*^ER^**: Once activated via its dimerization and autophosphorylation, IRE1α cleaves a select group of mRNAs and miRNAs to drive their degradation through a process known as regulated IRE1-dependent decay (RIDD), reducing the total protein folding load on the ER. IRE1α also facilitates the unconventional splicing of *XBP1* mRNA into its spliced form, a potent transcription factor known as XBP1s, which drives the expression of genes tied to protein quality control to restore ER homeostasis. *PERK-UPR*^ER^**: PERK also dimerizes and autophosphorylates upon ER stress, which then phosphorylates eIF2α to attenuate global translation. The mRNA of transcription factor *ATF4* is preferentially translated following eIF2α phosphorylation, allowing it to upregulate genes involved in amino acid metabolism, oxidative stress resistance, autophagy, and apoptosis. *ATF6-UPR*^ER^**: ER stress unmasks several Golgi-localization signals within ATF6 that allow it to translocate to the Golgi body. There, it is sequentially cleaved by site-1 protease (S1P) and site-2 protease (S2P) from its full-length form (ATF6p90) into its transcriptionally active form (ATF6p50), which initiates the transcription of UPR target genes pertaining to protein quality control and ER biogenesis to promote ER secretory capacity. Solid arrows represent direct actions.

Upon sensing ER stress, the second step of the UPR*^ER^* process is to resolve the homeostatic disruption. To facilitate this, the UPR*^ER^* is coordinated through three distinct transmembrane proteins that initiate parallel transcriptional and translational responses: inositol-requiring enzyme 1α (IRE1α), protein kinase RNA-like endoplasmic reticulum kinase (PERK) and activating transcription factor 6 (ATF6) (see Figure 1). Each protein contains a luminal stress-sensing domain within the ER, along with a cytosolic fragment that facilitates transduction of the stress signal to the nucleus. The sensors are typically kept dormant under homeostatic conditions through the binding of ER chaperone binding immunoglobulin protein (BiP) to their luminal domains. However, upon ER stress, BiP preferentially associates with misfolded or unfolded proteins rather than IREα, PERK, or ATF6 leading to activation of UPR*^ER^* signaling pathways (Bertolotti et al., 2000; Shen J. et al., 2005).

IRE1α, a protein kinase/endoribonuclease that was first identified in yeast as required for protection against ER stress, sits atop the most evolutionarily conserved arm of the UPR*^ER^* (hereafter: IRE1a-UPR*^ER^*) (Cox et al., 1993; Mori et al., 1993). Following its activation via dimerization and subsequent autophosphorylation, IRE1α processes the mRNA encoding X-box binding protein 1 (XBP1), removing a small intron that facilitates the translation of *XBP1* into its active form as a potent transcription factor (XBP1s) (Sidrauski and Walter, 1997; Shen et al., 2001; Yoshida et al., 2001; Calfon et al., 2002). XBP1s then translocates to the nucleus where it drives the induction of genes involved in protein trafficking, folding, and degradation (Lee et al., 2003a; Shen X. et al., 2005; Acosta-Alvear et al., 2007). XBP1s-induced protein degradation occurs largely through ER-associated degradation (ERAD), which targets ER-localized proteins for cytosolic degradation via the proteasome (Hwang and Qi, 2018). Independent of XBP1s activation, IRE1α also cleaves a subset of mRNAs and microRNAs (miRNAs) that leads to their rapid degradation through a process known as regulated IRE1-dependent decay (RIDD) (Hollien et al., 2009; Hollien and Weissman, 2006; Upton et al., 2012). ER-localized transcripts are preferentially targeted for degradation as a means to reduce the total translation and protein-folding burden placed on the ER (Maurel et al., 2014).

The second arm of the UPR*^ER^* is governed by the protein kinase PERK (hereafter: PERK-UPR*^ER^*). Like IRE1α, PERK undergoes dimerization and autophosphorylation following ER stress. Following its activation, PERK acts through its kinase domain to attenuate translation via the phosphorylation of eukaryotic initiation factor 2α (eIF2α) to prevent the influx of newly synthesized proteins into the ER (Harding et al., 1999). However, this translational shutdown also permits a select group of mRNA transcripts to undergo translation; one of which is activating transcription factor 4 (ATF4) (Lu et al., 2004; Vattem and Wek, 2004). Following activation, ATF4 induces the expression of genes tied to amino acid metabolism and oxidative stress resistance (Harding et al., 2003), but during persistent ER stress can also promote apoptotic signaling through an interaction with downstream transcription factor C/EBP homologous protein (CHOP) (Ma et al., 2002; Han et al., 2013). The ATF4/CHOP interaction also forms part of a negative feedback loop through upregulation of the protein phosphatase 1 (PP1) regulatory subunit GADD34, which forms a complex with PP1 to dephosphorylate eIF2α and restore translational capacity following the resolution of ER stress (Novoa et al., 2001).

The third arm of the UPR*^ER^* is regulated through ATF6 (hereafter: ATF6-UPR*^ER^*). Upon ER stress, BiP dissociates from ATF6, unmasking two Golgi-localization signals that cause ATF6 to translocate to the Golgi body (Shen et al., 2002). Here, the full-length form of ATF6, ATP6p90, is sequentially cleaved by site-1 protease (S1P) and site-2 protease (S2P), releasing the cytosolic ATF6p50 fragment that translocates to the nucleus to function as a transcription factor (Haze et al., 1999; Ye et al., 2000). ATF6p50 then induces the expression of genes pertaining to protein quality control and ER biogenesis to improve ER secretory capacity (Wu et al., 2007; Yamamoto et al., 2007; Bommiasamy et al., 2009). Of note, the transcriptional program induced by ATF6p50 displays a degree of overlap with that of XBP1s. Indeed, ATF6p50 and XBP1s exhibit crosstalk via the formation of heterodimers, likely serving to fine tune the transcriptional output following ER stress (Yamamoto et al., 2007; Shoulders et al., 2013).

Through the combined IRE1α/PERK/ATF6 signaling axes, the adaptive UPR*^ER^* phase represents a rapid response to promote protein quality control and allow the resumption of important cellular functions performed by the ER.

#### Non-cell Autonomous Endoplasmic Reticulum Unfolded Protein Response Signaling

A host of recent work in *Caenorhabditis elegans* has highlighted an additional novel aspect of UPR*^ER^* biology – the ability to communicate ER stress across multiple tissue types to coordinate a whole-body organismal response. Expression of *xbp-1s* specifically in worm neurons leads to UPR*^ER^* activation in the intestine and confers greater organismal resistance against ER stress (Taylor and Dillin, 2013). This method of intercellular communication relies on the release of small clear vesicles (SCVs) carrying neuronally derived stress signals to distal tissue (Taylor and Dillin, 2013). Intriguingly, expression of *xbp-1s* in glial cells elicits a similar non-cell autonomous mode of intestinal UPR*^ER^* activation, though via a different mechanism involving neuropeptide signaling (Frakes et al., 2020).

Further investigation of the non-cell autonomous mechanism in nematodes has revealed that neuronally expressed *xbp-1s* regulates intestinal stress resistance via several distinct methods, including the upregulation of lysosomal activity to improve proteostasis (Imanikia et al., 2019a) and alterations of lipid metabolism (Imanikia et al., 2019b; Daniele et al., 2020). Additionally, it was demonstrated that distinct neuronal populations apply these mechanisms differentially; dopaminergic *xbp-1s* expression drives lipid remodeling, while serotonergic *xbp-1s* expression induces chaperones to promote protein quality control (Higuchi-Sanabria et al., 2020). Finally, upon ER or starvation stress, *xbp-1* is spliced in two interneurons required for tyramine synthesis, which drives intestinal UPR*^ER^* activation and modulates changes in reproductive and feeding behavior (Özbey et al., 2020).

Taken together, these studies serve to highlight the mechanistic complexity of the UPR*^ER^*; stress signals can produce distinct responses depending on where in the CNS it is received, while a pan-neuronal response likely integrates multiple stress signals to drive a more comprehensive modulation of ER function to maintain organismal homeostasis. Importantly, distal UPR*^ER^* activation appears to be conserved through evolution; activation of *Xbp1s* in mouse proopiomelanocortin (Pomc) neurons regulates metabolic alterations through UPR*^ER^* activation in the liver (Williams et al., 2014; Brandt et al., 2018). Thus, the non-cell autonomous aspect of UPR*^ER^* signaling appears to play a key role in orchestrating the whole-body adaptive response to ER stress.

#### Acute Versus Chronic Endoplasmic Reticulum Unfolded Protein Response Activation

Should the adaptive phase of the UPR*^ER^* prove insufficient in restoring ER proteostasis, chronic UPR*^ER^* activation can drive cells toward an apoptotic fate. Much like the adaptive response, UPR*^ER^*-mediated apoptotic signaling consists of the integration of several interweaved pathways.

The canonical intrinsic apoptotic pathway, regulated by the B cell lymphoma 2 (Bcl2) family of proteins and characterized by the permeabilization of the mitochondrial outer membrane (MOM) and release of pro-apoptotic factors including cytochrome c (Pihán et al., 2017), can be initiated through separate means by both PERK and IRE1α-mediated signaling. PERK/ATF4-mediated activation of CHOP can inhibit the negative regulator of cell death Bcl2 (McCullough et al., 2001) and activate pro-apoptotic factor BIM (Puthalakath et al., 2007), with both outcomes converging on pro-apoptotic signaling through core Bcl2 proteins BAX and BAK (Pihán et al., 2017). Additionally, together with ATF4, CHOP can promote cell death by enhancing protein synthesis and reactive oxygen species (ROS) production under chronic ER stress (McCullough et al., 2001; Marciniak et al., 2004; Han et al., 2013).

Sustained IRE1α RNAse activity leads to the decay of a select group of precursor miRNAs that typically repress the translation of initiator protease Caspase-2 (Upton et al., 2012). This allows Caspase-2 to cleave BID and induce BAX/BAK-mediated apoptosis (Wei et al., 2001; Bonzon et al., 2006). However, a separate study did not observe Caspase-2 activation following ER stress, nor its requirement for ER stress-induced cell death (Sandow et al., 2014), suggesting that this mechanism requires further investigation. Under chronic ER stress, RIDD also induces thioredoxin-interacting protein (TXNIP) via the degradation of its negative regulator microRNA-17, leading to activation of the NLRP3 inflammasome and its subsequent downstream hallmarks, sterile inflammation and pyroptotic cell death (Lerner et al., 2012). Additionally, activated IRE1α can form a complex with adaptor protein TRAF2 and ASK1 to initiate a JNK-mediated signaling cascade that ultimately drives cell death (Urano et al., 2000; Nishitoh et al., 2002).

Both pro-survival and pro-death signals are integrated through the UPR*^ER^* to decide cell fate, but how this balance is tipped to favor a specific fate remains an important question in the field (Hetz et al., 2020). Indeed, UPR-induced apoptosis may prove beneficial under certain physiological contexts as the removal of damaged or dysfunctional cells can prevent the triggering of a detrimental inflammatory response (Tabas and Ron, 2011), and sustained ER stress can contribute to a range of pathological outcomes if left unresolved (Wang and Kaufman, 2016).

### Physiology of the Mitochondrial Unfolded Protein Response

#### Mechanism of the Mitochondrial Unfolded Protein Response

##### Mitochondrial Unfolded Protein Response Activation

Mitochondrial proteostasis failure is the most thoroughly studied trigger of the UPR*^mt^*, and it can arise from (1) accumulated misfolded proteins, (2) impaired mitochondrial translation, (3) an imbalance in the ratio of mitochondria-derived to nuclear-derived proteins in the mitochondria (hereafter: mito-nuclear imbalance), or (4) impaired mitochondrial protein import. Early work in the field using monkey kidney cells showed that misfolded mitochondrial proteins cause an increase in protease and chaperone transcript and protein levels (Zhao, 2002). Subsequent work in invertebrates and mammals has shown that loss of mitochondrial proteases such as LONP1 or mitochondrial chaperones mtHSP70, HSP60, or TRAP1 causes accumulation of misfolded proteins and the activation of the UPR*^mt^* (Yoneda et al., 2004; Münch and Harper, 2016). Impaired mitochondrial translation can also activate the UPR*^mt^*, likely due to reduced protein levels and the accumulation of truncated proteins (Dogan et al., 2014; Chung et al., 2017). Another proteostasis-related trigger of the UPR*^mt^* is mito-nuclear imbalance, which impairs the ability for multi-protein complexes to form creating unfolded protein stress (Houtkooper et al., 2013; Mouchiroud et al., 2013; Yuan et al., 2020). Lastly, impaired mitochondrial protein import functions as perhaps the major sensor activating the UPR*^mt^* because mitochondrial protein import requires ATP, mtHSP70, and an intact mitochondrial membrane potential and thus is rapidly perturbed in response to most mitochondrial stresses, however minor (Shpilka and Haynes, 2018). This has been demonstrated in *C. elegans* or human cell lines; knockdown of *TIM23*, which encodes a major translocase located on the mitochondrial inner membrane (MIM), reduces protein import and activates the UPR*^mt^* (Rainbolt et al., 2013).

Electron transport chain (ETC) dysfunction can also initiate the UPR*^mt^*. Mild inhibition of genes encoding mitochondrial ETC components in *C. elegans* activates the UPR*^mt^* transcriptional regulators atfs-1, dve-1, and ubl-5 (Haynes et al., 2007; Nargund et al., 2012). Furthermore, decreasing the expression of mitochondrial ETC complex subunits or reducing ETC complex function has been shown to induce the expression of UPR*^mt^*-related chaperones in *C. elegans*, *Drosophila*, and mammals (Yoneda et al., 2004; Hamilton et al., 2005; Benedetti et al., 2006; Owusu-Ansah et al., 2013; Pulliam et al., 2014). Additionally, a mito-nuclear imbalance of ETC proteins can result in these unfolded proteins accumulating within the mitochondrial matrix and activating the UPR*^mt^* (Martinus et al., 1996; Yoneda et al., 2004; Nargund et al., 2012; Houtkooper et al., 2013; Liu et al., 2014).

Lastly, changes in mtDNA can also induce the UPR*^mt^*. Loss of mtDNA increases expression of nuclear-encoded mitochondrial chaperones but not cytosolic chaperones suggesting an activation of the UPR*^mt^* (Martinus et al., 1996). Additionally, harboring even a small amount of damaged mtDNA is sufficient to induce the UPR*^mt^* (Gitschlag et al., 2016; Lin et al., 2016). Furthermore, mutations in mtDNA helicases increase the expression of UPR*^mt^*-related proteins suggesting that changes to the structure of mtDNA, in addition to the sequence of mtDNA, can activate the UPR*^mt^* (Yoneda et al., 2004; Khan et al., 2014; Forsström et al., 2019).

##### Mitochondrial Unfolded Protein Response Effectors and Effects

**FIGURE 2:**
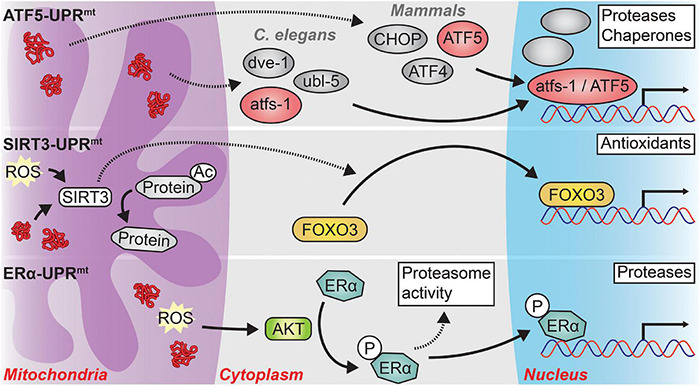
The three major signaling pathways of the UPR*^mt^*. In response to mitochondrial stress, three distinct branches of the UPR*^mt^* may be activated, depending on the type and location of the mitochondrial stress. *ATF5-UPR*^mt^**: In *C. elegans*, protein stress in the mitochondrial matrix causes the cytosolic accumulation of atfs-1, or its mammalian ortholog ATF5. In concert with the transcription factor dve-1 and the ubiquitin-like protein ubl-5, atfs-1 translocates to the nucleus where it induces the transcription of proteases and chaperones to relieve mitochondrial protein stress. A similar process occurs in mammals, albeit with the requirement of two transcription factors, CHOP and ATF4, in addition to ATF5. The precise interactions among atfs-1, dve-1, and ubl-5 as well as among ATF5, CHOP, and ATF4 remain unclear. *SIRT3-UPR*^mt^**: Mitochondrial matrix reactive oxygen species (ROS) or protein stress activates SIRT3 which then directly deacetylates numerous mitochondrial proteins and indirectly causes the nuclear localization of the transcription factor FOXO3. FOXO3 then induces an antioxidant transcriptional program to combat high levels of oxidative stress in the mitochondria. *ERα-UPR*^mt^**: Misfolded proteins and ROS located within the mitochondrial intermembrane space (IMS) activate the kinase AKT. AKT phosphorylates Estrogen Receptor alpha (ERα) which then increases the activity of the proteasome and functions as a transcription factor in the nucleus to induce the expression of IMS-specific proteases. Solid arrows represent direct actions while dashed arrows represent indirect actions or actions with unclear mechanisms.

There are three parallel – and potentially overlapping – mechanisms linking mitochondrial stresses to nuclear transcriptional responses relevant to aging and neurodegeneration (see Figure 2). The classical UPR*^mt^* mechanism, primarily researched in worms and subsequently shown to be highly conserved in mammals, is primarily driven by the nuclear localization of the transcription factor atfs-1 in worms or the transcription factors CHOP, ATF4, and ATF5 in mammals (hereafter: ATF5-UPR*^mt^*) (Shpilka and Haynes, 2018). Two other arms of the UPR*^mt^* have been shown to be at least partially independent of the classical UPR*^mt^* axis. One signals through the mitochondrial NAD^+^-dependent deacetylase SIRT3 and the other is dependent upon the activation of the hormone receptor Estrogen Receptor alpha (ERα) (Papa and Germain, 2011, 2014). Activation of either of these alternative UPR*^mt^* arms induces differing cellular effects from those of the classical arm of the UPR*^mt^*.

The most thoroughly studied arm of the UPR*^mt^* is the ATF5-UPR*^mt^*. In both worms and mammals, the ATF5-UPR*^mt^* modulates both transcription and chromatin organization to counter mitochondrial stress (Haynes et al., 2010; Nargund et al., 2012; Fiorese et al., 2016). In *C. elegans*, atfs-1, which contains a nuclear localization sequence and a mitochondrial targeting sequence, is normally imported into the mitochondria and rapidly degraded by the mitochondrial matrix protease lonp-1 (Haynes et al., 2010; Nargund et al., 2012, 2015). However, under conditions of mitochondrial stress, import of atfs-1 into the mitochondria is precluded, and atfs-1 is trafficked to the nucleus where it coordinates with other factors, including ubl-5 and dve-1, to elicit the UPR*^mt^* transcriptional program (Benedetti et al., 2006; Haynes et al., 2007, 2010; Nargund et al., 2012, 2015). In mammals, ATF5 plays a similar role to that of atfs-1; however, the transcription factors CHOP and ATF4 are also required for the induction of the UPR*^mt^*, although it remains unclear precisely how they interact to do so (Aldridge et al., 2007; Horibe and Hoogenraad, 2007; Fiorese et al., 2016; Quirós et al., 2017).

In addition to transcriptional changes, the ATF5-UPR*^mt^* modifies the epigenetic landscape to promote an open chromatin state at UPR*^mt^* genes and repress transcription of genes antagonistic to the UPR*^mt^*. In *C. elegans*, the ATF5-UPR*^mt^* promotes global heterochromatin formation through the methylation of H3K9 by met-2 and the nuclear co-factor lin-65 (Andersen and Horvitz, 2007; Tian et al., 2016). Despite this global chromatin silencing, other chromatin regions become more open, in part due to stabilization by the transcription factor dve-1 (Tian et al., 2016). Simultaneously, the demethylases jmjd-1.2 and jmjd-3.1, or the mammalian equivalents PHF8 and KDM6B, create transcriptionally active chromatin marks in a process that is dependent upon CREB binding protein 1 (cbp-1), the ortholog of the mammalian CBP/p300 (Merkwirth et al., 2016; Li et al., 2021).

For invertebrates as well as mammals, the translational and epigenetic changes induced by the mitochondrial stress signaled through the ATF5-UPR*^mt^* result in (1) an upregulation of the expression of mitochondrial chaperones, proteases, protein importers, and ETC components; (2) a reduction in global protein translation in the cytosol; (3) a decrease in protein translation within mitochondria; and (4) a reprogramming of mitochondrial metabolism (Zhao, 2002; Yoneda et al., 2004; Aldridge et al., 2007; Baker et al., 2012; Nargund et al., 2012, 2015; Houtkooper et al., 2013; Gitschlag et al., 2016; Münch and Harper, 2016; Borch Jensen et al., 2018; Molenaars et al., 2020; Yuan et al., 2020). Cumulatively, these effects reduce proteotoxic stress in the mitochondrial matrix by increasing protein folding and degradation capacity, reducing protein burden within the mitochondria, and shifting metabolic demand away from the mitochondria, presumably to allow for the restoration of proper ETC function.

As opposed to the ATF5-UPR*^mt^*, which responds primarily to proteotoxic stress within the mitochondrial matrix, the ERα arm of the UPR*^mt^* (hereafter: ERα-UPR*^mt^*) responds to proteotoxic stress within the mitochondrial inner membrane space (IMS) (Radke et al., 2008; Papa and Germain, 2011). Proteotoxic stress and ROS within the IMS leads to the activation ERα in an AKT-dependent fashion (Papa and Germain, 2011). ERα increases the activity of the cytosolic ubiquitin-proteasome system and translocates to the nucleus where it functions as a transcription factor to induce the expression of IMS-specific proteases such as *HTRA2*; both processes reduce protein stress in the mitochondrial IMS (Radke et al., 2008; Papa and Germain, 2011). Interestingly, it appears that if the ERα-UPR*^mt^* is not working, the ATF5-UPR*^mt^* will eventually be activated (Papa and Germain, 2011). This suggests that there may be direct spillover of protein stress from the IMS to the matrix to trigger the ATF5-UPR*^mt^*.

The last arm of the UPR*^mt^* signals through SIRT3 and the transcription factor FOXO3 (hereafter: SIRT3-UPR*^mt^*) (Papa and Germain, 2014; Marcus and Andrabi, 2018). SIRT3-UPR*^mt^* signaling occurs both by SIRT3-dependent deacetylation of mitochondrial targets and SIRT3/FOXO3-dependent transcriptional changes (Schwer et al., 2002; Lombard et al., 2007; Sundaresan et al., 2009). The SIRT3/FOXO3-dependent signaling induces an antioxidant transcriptional program to combat high levels of ROS within the mitochondria (Papa and Germain, 2014; Marcus and Andrabi, 2018). As an example of the dual nature of this arm, expression of misfolding-prone proteins or pharmacological inhibition of ETC complexes causes a ROS-dependent activation of SIRT3 which then deacetylates a number of downstream targets leading to the nuclear-localization of FOXO3 (Papa and Germain, 2014).

Despite significant overlap between the ATF5-UPR*^mt^* and SIRT3-UPR*^mt^*, there is significant evidence that the SIRT3-UPR*^mt^* is a *bona fide* unique and parallel arm of the UPR*^mt^* that is separable from the ATF5-UPR*^mt^* (Münch, 2018). CHOP, a transcription factor required for the mammalian ATF5-UPR*^mt^*, is dispensable for the expression of antioxidants in response to mitochondrial stress (Papa and Germain, 2014). Additionally, ETC complex I and III inhibitors – known to increase levels of mitochondrial ROS (mtROS) – increase levels of SIRT3 and FOXO3 suggesting that ROS are sufficient to induce the SIRT3-dependent UPR*^mt^* (Papa and Germain, 2014).

The overlap between the ATF5-UPR*^mt^* and SIRT3-UPR*^mt^* likely arises due to the multiple actions of ROS within the mitochondria. It is likely that ROS *per se* activates the SIRT3-UPR*^mt^* while ROS-dependent protein damage and aggregation stimulates the ATF5-UPR*^mt^*. In this way, the SIRT3-UPR*^mt^* is the primary response to imbalances in redox homeostasis within the mitochondria while the ATF5-UPR*^mt^* is activated in circumstances where elevated ROS levels are too high or persist too long causing oxidative damage. Furthermore, the SIRT3-UPR*^mt^* appears to maintain ROS within a homeostatic window and allow for critical ROS-dependent physiological signaling to occur without excess oxidative damage occurring due to promiscuous ROS interactions with mitochondrial macromolecules (Kenny and Germain, 2017). Indeed, as evidence of such, a transient increase in mtROS results in the persistent, steady-state decrease in ROS levels 48-h later (Schmeisser et al., 2013). This suggests that mtROS spikes induce the expression of antioxidant machinery that installs a new redox homeostasis setpoint and reduces total steady-state levels of mtROS.

#### Non-cell Autonomous Mitochondrial Unfolded Protein Response Signaling

Similar to the non-cell autonomous effect observed with the UPR*^ER^*, the induction of the UPR*^mt^* also exerts non-cell autonomous effects on distant tissues. In *C. elegans*, signaling pathways dependent upon the Wnt ligand egl-20, the neuropeptide flp-2, the neurotransmitter serotonin, and the G-protein coupled receptor fshr-1 have been linked to non-cell autonomous signaling in response to neuronal mitochondrial dysfunction (Berendzen et al., 2016; Shao et al., 2016; Zhang et al., 2018; Kim and Sieburth, 2020). It also appears to be the case in mammals that secreted factors, termed mitokines, relay mitochondrial stress to distant tissues (Durieux et al., 2011). Numerous mitochondrial stresses from impaired protein translation to mtDNA deletions can be signaled to distant tissues through the secretion of FGF21 and GDF15 (Suomalainen et al., 2011; Chung et al., 2017; Forsström et al., 2019; Kang et al., 2021). In many cases, however, it remains unclear exactly how these individual mitokines induce the wide-ranging transcriptional changes characteristic of the UPR*^mt^*. It appears that much of the non-cell autonomous signaling of mitochondrial dysfunction originates from neurons, perhaps because neuronal mitochondria, as hubs of metabolism in a metabolically sensitive cell population, function as sentinels of metabolic stress to prime the whole organism for a high stress environment (Durieux et al., 2011; Tian et al., 2016). However, it should be noted that non-neuronal tissues such as gonads or muscles also have a role in non-cell autonomous UPR*^mt^* signaling which underscores the usefulness of a rapidly inducible transcriptional program that can modulate the capacity of cellular mitochondria to engage in energy production, biosynthesis, calcium homeostasis, and immune signaling (Owusu-Ansah et al., 2013; Lan et al., 2019).

#### Acute Versus Chronic Mitochondrial Unfolded Protein Response Activation

Like with the UPR*^ER^*, acute activation of the UPR*^mt^* results in distinct outcomes from that of chronic UPR*^mt^* activation. The UPR*^mt^* can induce acute, pro-survival transcriptional and epigenetic changes that alleviate the inciting mitochondrial stress (Münch, 2018). However, in cases where the instigating stress cannot be resolved, a prolonged response occurs that can cause detrimental outcomes such as reduced fecundity and the propagation of deleterious mtDNA (Rauthan et al., 2013; Gitschlag et al., 2016; Lin et al., 2016). Experimental approaches seem to offer conflicting evidence for whether chronic UPR*^mt^* is beneficial or detrimental for organismal fitness. Whereas some reports suggest mild, constitutive RNAi knockdown of components of the ETC extends lifespan in fruit flies, others suggest that partial inactivation of ETC genes does not extend lifespan (Rera et al., 2010; Owusu-Ansah et al., 2013). While knockdown of ETC complex subunits is a known trigger of the UPR*^mt^*, it remains possible that other mechanisms account for the differences in organism longevity in these studies. However, what is apparent is that long-term hyperactivation of the UPR*^mt^* is detrimental for both cellular and organismal health (Rauthan et al., 2013; Ishizawa et al., 2019). This is perhaps best illustrated by the lack of genetic mutations that exist that chronically activate the UPR*^mt^*; these mutations presumably have evolutionary trade-offs that, in the long-term, favor neither the cell nor the organism (Lamech and Haynes, 2015). That being said, it does appear that some low basal level of UPR*^mt^* activation is required for proper cellular health, perhaps as a buffer against subtle forms of mitochondrial stress (Shpilka and Haynes, 2018).

## The Unfolded Protein Response in Aging

It has become increasingly apparent from research conducted over the past few years that aging, the functional decline of biological systems that occurs over time eventually leading to both cellular and organismal dysfunction and death, is driven significantly by the loss of proteostasis and mitochondrial dysfunction (López-Otín et al., 2013; Hipp et al., 2014; Piper and Partridge, 2018; Singh et al., 2019). However, it remains unclear what role the UPR*^ER^* and UPR*^mt^*, as responses to age-dependent proteostasis collapse and mitochondrial dysfunction, play in compensating for these aging-related changes to preserve cellular function and organismal fitness. Here, we discuss how the functionality of the UPR*^ER^* and UPR*^mt^* are affected with age and how manipulations targeting the UPR*^ER^* and UPR*^mt^* can influence organismal aging in model systems.

### The Endoplasmic Reticulum Unfolded Protein Response in Aging

#### Changes to the Endoplasmic Reticulum Unfolded Protein Response in Aging

Studies in several model systems have provided evidence of changes to the UPR*^ER^* with aging. Work in *C. elegans* has demonstrated that the ability to respond to ER stress through the UPR*^ER^* is not only weakened with age, but that this decline also appears to be an early event during the aging process (Ben-Zvi et al., 2009; Taylor and Dillin, 2013). ER-localized protein chaperones, including BiP and protein disulfide isomerase (PDI), undergo oxidative damage with age in mouse liver, contributing to the age-associated increase in misfolded protein products within the ER (Rabek et al., 2003; Nuss et al., 2008). Within the aged mouse brain, the ability to induce adaptive UPR*^ER^* signaling following sleep deprivation is diminished, while evidence of dysregulated UPR*^ER^* homeostasis with age is also observed in two neuronal subpopulations, orexinergic and noradrenergic neurons, that govern wakefulness (Naidoo et al., 2008, 2011). In aged rat brains, induction of the IRE1α-UPR*^ER^* and the ATF6-UPR*^ER^* is suppressed following proteasome inhibition, coinciding with elevated levels of pro-apoptotic factors CHOP, BAX, and BAK (Paz Gavilán et al., 2009). A similar pattern is also observed in other tissues from aged rats; lung, liver, kidney, and spleen all experience loss of ATF4 and BiP, whilst CHOP and phosphorylated JNK are elevated (Hussain and Ramaiah, 2007). Finally, a recent *in vitro* study demonstrated that senescent human lung fibroblasts remain capable of sensing ER stress effectively but lose the ability to coordinate the subsequent transcriptional response through XBP1s and ATF6 (Sabath et al., 2020). Collectively, these studies show that the UPR*^ER^* loses the ability to mount the adaptive response with age. In contrast, pro-apoptotic signaling potency is retained, partly due to a lowering of the homeostatic threshold that determines the switch from acute to chronic UPR*^ER^* signaling in aging cells.

#### Manipulations to the Endoplasmic Reticulum Unfolded Protein Response Affect Aging

The established decline of the UPR*^ER^* with age suggests that manipulations aimed at maintaining adaptive UPR*^ER^* functionality throughout the organism’s lifespan may prove beneficial to healthy aging. Indeed, a study on long-lived naked mole rats uncovered protein quality control maintenance as a key determinant of healthy aging (Pérez et al., 2009b). In this regard, model organisms have provided excellent experimental systems to dissect the role of UPR*^ER^* signaling in aging.

##### Inositol-Requiring Enzyme 1α-Endoplasmic Reticulum Unfolded Protein Response in Aging

The most conserved pathway within the UPR*^ER^*, IRE1α/XBP1 signaling has been shown to regulate aging from yeast to mammals. IRE1α and yeast XBP1 homolog HAC1 are both required for yeast replicative lifespan extension following several UPR*^ER^* manipulations, either through the specific deletion of UPR*^ER^* target genes which leads to constitutive UPR*^ER^* signaling (Labunskyy et al., 2014), or via the enhancement of basal UPR*^ER^* activity (Cui et al., 2015). In nematodes, constitutive neuronal, glial, or intestinal expression of *xbp-1s* restores UPR*^ER^* functionality with age and is sufficient to extend lifespan (Taylor and Dillin, 2013; Frakes et al., 2020). Of note, ubiquitous expression of *xbp-1s* does not extend lifespan, while its expression specifically in muscle cells decreases lifespan (Taylor and Dillin, 2013), suggesting that constitutive XBP1s activity can prove detrimental in certain tissues.

The IRE1α-UPR*^ER^* also influences organismal lifespan in conjunction with other well-established aging pathways. Both IRE1α and XBP1 are required for enhanced longevity through the reduction of the insulin/IGF-1 like signaling (IIS) pathway, whereby xbp-1 coordinates with daf-16, the *C. elegans* ortholog of FOXO3, to upregulate genes involved in longevity and ER stress resistance (Henis-Korenblit et al., 2010). In yeast, HAC1 activity is necessary for lifespan extension via dietary restriction (DR) (Choi et al., 2013). The IRE1α-UPR*^ER^* also contributes to dietary-induced lifespan modulation in nematodes, where it is required for the longevity effects observed under both DR conditions and in *hypoxia-inducible factor-1* (*hif-1*) mutants under high nutrient conditions (Chen et al., 2009). Additionally, IRE1α is essential for DR-induced lifespan extension in conjunction with transcription factor pha-4, whereby DR in early life promotes ERAD function to improve proteostasis with age (Matai et al., 2019). Finally, low-nutrient conditions activate xbp-1 in tyramine-producing neurons to alter reproductive and feeding behavior in parallel with intestinal UPR*^ER^* activation to extend lifespan (Özbey et al., 2020). Of note, Xbp1 can also promote distal UPR*^ER^*-mediated metabolic adaptation following food sensing in fruit flies (Luis et al., 2016) and mice (Williams et al., 2014; Brandt et al., 2018), highlighting the evolutionary conservation of this nutrient sensing role for the IRE1α-UPR*^ER^*. Dietary interventions have been well-established as modulators of organismal health with age (Fontana and Partridge, 2015), and evidence garnered in model organisms has placed the UPR*^ER^*, in particular the IRE1α-UPR*^ER^*, as a key signaling pathway within this form of lifespan regulation.

##### Protein Kinase RNA-Like Endoplasmic Reticulum Kinase-Endoplasmic Reticulum Unfolded Protein Response in Aging

Dysregulation of the PERK-UPR*^ER^* is a common feature with age (Hussain and Ramaiah, 2007; Paz Gavilán et al., 2009; Sabath et al., 2020), and manipulations targeting this pathway have focused mainly on suppressing aberrant PERK signaling. Early work observed that genetic deletion of the nematode PERK ortholog *pek-1* does not affect lifespan (Henis-Korenblit et al., 2010). In contrast, a more recent study demonstrated that a single amino acid substitution in the pek-1 kinase domain prolongs lifespan (Derisbourg et al., 2021), which may reflect the difference in *pek-1* mutations between studies. In addition, abolishing the activation of eIF2α via a phospho-deficient mutation also confers lifespan extension (Derisbourg et al., 2021), suggesting that downstream targeting of the PERK pathway can also promote longevity.

Work in *Drosophila* has demonstrated that ER stress triggers intestinal expression of the *Drosophila* PERK ortholog *Pek* via both cell autonomous and non-cell autonomous mechanisms, which then regulates intestinal stem cell (ISC) regeneration to maintain proliferative homeostasis (Wang et al., 2015). However, sustained Pek activity in ISCs, as seen in aged flies, shortens lifespan through gut dysplasia, serving to highlight the differential outcomes on lifespan attained between acute and chronic PERK signaling (Wang et al., 2015). In mice, reducing PERK activity can also prove beneficial; knockdown of *Perk* in the CA1 hippocampus region in aging mice reverses age-related declines in memory and neuronal excitability (Sharma et al., 2018). However, the outcome of neuronal PERK inhibition in mammals appears cell-specific; deletion of *Perk* specifically in mouse dopaminergic neurons leads to a range of both cognitive and motor phenotypes via the dysregulation of *de novo* translation and dopamine release (Longo et al., 2021). Taken together, these murine studies demonstrate the complexity involved in inhibiting PERK activity; reducing PERK function has the potential to affect both the beneficial acute and deleterious chronic modes of signaling. Indeed, the long-lived *C. elegans* phospho-deficient *eIF2α* mutant was not impaired in either global translation capacity or ER stress resistance, suggesting that the fine-tuning of PERK signaling, rather than ablation, has the potential to slow aging (Derisbourg et al., 2021).

##### Activating Transcription Factor 6-Endoplasmic Reticulum Unfolded Protein Response in Aging

The ATF6-UPR*^ER^* is the least studied branch of the UPR*^ER^* in the context of aging. Like the IRE1α-UPR*^ER^*, evidence suggests that the ATF6-UPR*^ER^* loses adaptive signaling potency with age (Paz Gavilán et al., 2009; Sabath et al., 2020), and deletion of *Atf6* renders mice sensitive to sources of chronic ER stress (Wu et al., 2007; Yamamoto et al., 2007). However, recent work in nematodes suggests that the relationship between the ATF6-UPR*^ER^* and age is more complex. Burkewitz et al. (2020) demonstrated that atf-6 in *C. elegans* is dispensable for resistance to proteotoxic stress, and that its loss confers longevity via the downregulation of its conserved transcriptional target calreticulin, a regulator of ER calcium homeostasis. This result is in apparent contradiction with the perceived role of ATF6 as a largely protective UPR*^ER^* effector. Together with the observation that calreticulin is elevated with age in an atf6-dependent manner, this study suggests that ATF6 signaling can prove detrimental with age under basal conditions, independent of its canonical UPR*^ER^* role in maintaining proteostasis (Burkewitz et al., 2020).

#### Non-cell Autonomous Endoplasmic Reticulum Unfolded Protein Response Signaling and Aging

Given the recently uncovered importance of non-cell autonomous UPR*^ER^* signaling in regulating whole-body organismal homeostasis, it is no surprise that this mechanism also plays a role in the aging process. In *C. elegans*, neuronal *xbp-1s* expression leads to intestinal UPR*^ER^* activation, conferring extended lifespan and improved ER stress resistance with age (Taylor and Dillin, 2013). However, if distal communication to the intestine is disrupted via the intestinal knockdown of *xbp-1s* or inhibition of neuronal secretory regulator *unc-13*, the beneficial effects on longevity and stress resistance are lost (Taylor and Dillin, 2013). Similarly, the enhancements to lifespan and proteostasis attained via glia-intestine non-cell autonomous UPR*^ER^* signaling are both abolished in worms defective in neuropeptide release (Frakes et al., 2020). The specific messenger molecules required for non-cell autonomous UPR*^ER^* activation in nematodes, however, remain unknown in both the neuronal and glial paradigms. Identifying these mediators, and whether they themselves are conserved, are vital steps in uncovering how UPR*^ER^* regulates aging in a whole-body manner. Additionally, recent studies in mice have provided initial evidence of mammalian UPR*^ER^* signaling across distal tissues; *Xbp1s* expression in Pomc neurons drives UPR*^ER^* activation in the liver to promote metabolic homeostasis, protecting mice from diet-induced obesity (Williams et al., 2014; Brandt et al., 2018).

In line with these observations, the intestinal pathways initiated downstream of neuronal UPR*^ER^* signaling are also required to promote healthy aging. Impairing these UPR*^ER^*-mediated changes, namely increased lysosomal activity and lipid remodeling, block the lifespan extension conferred by neuronal *xbp-1s* expression (Imanikia et al., 2019a,b; Daniele et al., 2020). Interestingly, neuronal or intestinal expression of *xbp-1s* also enhances lysosome activity and proteostasis in distal muscle cells, while expression of *xbp-1s* specifically in muscle cells leads to a downregulation of lysosomal genes and reduced lifespan (Imanikia et al., 2019a). Thus, via modulation of two critical ER functions, protein and lipid homeostasis, distal UPR*^ER^* signaling can, in turn, benefit organismal health. Additionally, these findings also position the intestine as a critical regulator of organism-wide aging. This is in agreement with a host of recent studies demonstrating the role of the gut, in particular gut microbiota, as a key regulator of organismal aging (Smith et al., 2017; Donaldson et al., 2020; Boehme et al., 2021; Shukla et al., 2021). Whether gut microbial composition contributes to the non-cell autonomous UPR*^ER^* regulation of aging is at this stage largely unknown and provides an important target for investigation moving forward.

### The Mitochondrial Unfolded Protein Response in Aging

#### Changes to the Mitochondrial Unfolded Protein Response in Aging

Through observational investigations, activation of the UPR*^mt^* has been found to correlate positively with longevity across the animal kingdom. Experiments conducted in *C. elegans* have shown that the expression level of mitochondrial chaperones and proteases progressively increases across the lifespan and further studies have shown that worms with higher levels of basal UPR*^mt^* activation have longer lifespans (Sheng et al., 2021; Zhang et al., 2021). In mammals, analyses of cell cultures derived from long-lived mouse strains show upregulation of the UPR*^mt^* genes *Hsp60* and *Lonp1* (Ozkurede and Miller, 2019). Furthermore, Houtkooper and colleagues, by analyzing the natural variation of gene transcript levels in multiple strains of an inbred population of mice, show that conditions known to activate the UPR*^mt^* are correlated with longevity (Houtkooper et al., 2013). In total, these data suggest that the activation of the UPR*^mt^* with age is beneficial and extends lifespan.

However, data also exist suggesting that activation of the UPR*^mt^* is not correlated with lifespan extension. Higher plasma concentrations of the UPR*^mt^*-inducing mitokines FGF21 and GDF15 are associated with shorter lifespans, poorer handgrip strength, and increased insulin insensitivity in elderly adults (Conte et al., 2019). These plasma mitokines may represent a response to increased mitochondrial stress; however, the UPR*^mt^*-promoting effects of these mitokines may not be enacted due to decreased age-related inducibility of the UPR*^mt^* leading to a failure to alleviate the inciting mitochondrial stress and restore cellular homeostasis (Sheng et al., 2021).

#### Manipulations to the Mitochondrial Unfolded Protein Response Affect Aging

Direct manipulations of mitochondrial stress and components of the UPR*^mt^* have shed important insights into the role that UPR*^mt^* activation plays in aging. Overall, it appears that signaling through the ATF5-UPR*^mt^* and SIRT3-UPR*^mt^*, either independently, together, or in concert with other cellular pathways, slows aging and extends organismal lifespan.

##### Activating Transcription Factor 5-Mitochondrial Unfolded Protein Response in Aging

There is a large body of literature supporting the assertion that the transcriptional changes induced by the ATF5-UPR*^mt^* work to promote longevity and healthy aging across species. Multiple studies have shown that the lifespan extension observed in numerous mitochondrial ETC mutants is dependent upon signaling through atfs-1 or its downstream factors ubl-5 and dve-1 (Durieux et al., 2011; Wu et al., 2018; Gao et al., 2019). Impairment of mitochondrial protein translation extends *C. elegans* lifespan via a mechanism that is dependent upon the creation of a mito-nuclear imbalance within the mitochondria and signaling through the ATF5-UPR*^mt^*; this mechanism also appears to be conserved in mice (Houtkooper et al., 2013). Furthermore, as evidence of ATF5-UPR*^mt^*-dependent healthy aging, Owusu-Ansah and colleagues showed that flies with impaired ETC complex I function retain more motor function as they age compared to controls; this delay in age-related functional decline is partially lost upon knockdown of ATF5-UPR*^mt^*-dependent protease and chaperones (Owusu-Ansah et al., 2013).

However, data do exist that challenge the notion that ATF5-UPR*^mt^*-dependent transcriptional changes alter longevity. A critical study from the Kaeberlein lab provides multiple pieces of data that suggest that the ATF5-UPR*^mt^* is not involved in lifespan extension. They show that the activation of the UPR*^mt^* in the absence of mitochondrial stress does not extend lifespan in *C. elegans* and that atfs-1 is neither necessary nor sufficient for lifespan extension (Bennett et al., 2014). Collectively, these are surprising results, but the possibility remains that the pro-longevity effects of UPR*^mt^* activation are due to non-atfs-1-dependent mechanisms; that is, UPR*^mt^*-induced lifespan extension is separable from atfs-1-induced transcriptional changes. Indeed, studies that show atfs-1 playing a role in UPR*^mt^*-dependent lifespan extension only show a partial reduction in longevity upon deletion or knockdown of *atfs-1*; this suggests the involvement of other UPR*^mt^* or non-UPR*^mt^* pathways in promulgating mitochondrial stress-induced lifespan extension (Owusu-Ansah et al., 2013; Wu et al., 2018). Additionally, a recent study showed that knockdown of the ATP synthase component *atp-3* in early adulthood causes atfs-1-dependent activation of the UPR*^mt^* and reduces lifespan through a mechanism dependent upon the formation of a mitochondrial permeability transition pore (mPTP), a non-specific pore in the MIM that allows for increased movement of contents between the mitochondrial matrix and IMS (Angeli et al., 2021). Manipulations that prevent the formation of the mPTP restore lifespan and partially ameliorate the UPR*^mt^* activation (Angeli et al., 2021). It may be that the persistent opening of the mPTP in the context of *atp-3* manipulation is a strong pathological event that counteracts any beneficial effects of UPR*^mt^* activation (Pérez and Quintanilla, 2017). Lastly, some data suggest that genetic manipulations that induce mitochondrial stress do not extend lifespan (Durieux et al., 2011; Bennett et al., 2014). Thus, the lack of evidence of the universality of ATF5-UPR*^mt^*-induced lifespan extension points to the complexity of the relationship between ATF5-UPR*^mt^*-related cellular manipulations and organismal lifespan.

Epigenetic modifications induced by the ATF5-UPR*^mt^* seem to promote longevity. Numerous epigenetic modulators have been shown to play roles in maintaining the activation of the UPR*^mt^* and facilitating lifespan extension from worms to humans. These include the histone demethylases jmjd-1.2/PHF8, jmjd-3.1/KDM6B, the epigenetic reader baz-2/BAZ2B, the histone methyltransferase set-6/EHMT1, the acetyltransferase cbp-1/CBP/p300, and the histone deacetylase hda-1 (Merkwirth et al., 2016; Shao et al., 2020; Yuan et al., 2020; Li et al., 2021). Additionally, overexpression of histone H4 is sufficient to extend lifespan in *C. elegans* (Sural et al., 2020). To our knowledge, there exists no data showing that epigenetic changes associated with activation of the UPR*^mt^* are detrimental for longevity. In conclusion, the long-lasting induction of the UPR*^mt^* after the resolution of mitochondrial stress points to the existence of epigenetic changes that maintain the UPR*^mt^*-induced transcriptional state to induce lifespan extension (Durieux et al., 2011; Zhang et al., 2021).

##### ERα-Mitochondrial Unfolded Protein Response in Aging

Of the three identified arms of the UPR*^mt^*, the ERα-UPR*^mt^* arm is least implicated in longevity modulation. Evidence exists that administration of 17α-estradiol, which acts through ERα, can extend lifespan and that these effects are partly mediated through metabolic changes; however, it is yet to be shown whether these effects are mediated by the same ERα signaling pathways that are also activated by mitochondrial stress (Strong et al., 2016; Stout et al., 2017; Garratt et al., 2018; Mann et al., 2020). Additionally, increased expression of ERα appears to mediate some of the effects of lifespan extending manipulations such as caloric restriction (Yaghmaie et al., 2005; Morselli et al., 2014). It should be noted that multiple studies of the role of UPR*^mt^* activation on longevity have demonstrated marked reproductive changes with UPR*^mt^* activation (Dillin et al., 2002; Rea et al., 2007; Durieux et al., 2011; Baqri et al., 2014; Zhang et al., 2021). While not explicitly linked to ERα, there is certainly a relationship, however unclear, among UPR*^mt^* activation, lifespan extension, and reproductive capacity.

##### SIRT3-Mitochondrial Unfolded Protein Response in Aging

The SIRT3-UPR*^mt^* appears to play a role in lifespan extension, but whether these effects are due to direct SIRT3-dependent deacetylation of mitochondrial proteins or SIRT3/FOXO3-dependent transcriptional changes is not yet clear. *SIRT3* was first suggested as a longevity gene after the identification of multiple single nucleotide polymorphisms within *SIRT3* that are associated with lifespan extension (Hurst et al., 2002; Rose et al., 2003; Bellizzi et al., 2005). Later mechanistic studies have converged on Sirt3-dependent deacetylation of mitochondrial proteins that are involved in metabolism, proteostasis, and antioxidant defense as critical for lifespan extension in mammals (Hebert et al., 2013). Additionally, Sirt3 post-transcriptionally modifies the key UPR*^mt^* effectors Hsp10 and Lonp1; however, it is yet unclear whether this post-transcriptional modification plays a role in longevity modulation (Gibellini et al., 2014; Lu et al., 2015). While some data suggest that the deacetylation of mitochondrial proteins by SIRT3 may play a role in lifespan extension, it is entirely unclear whether the FOXO3-dependent transcriptional axis of the SIRT3-UPR*^mt^* is involved in controlling lifespan. Although nuclear accumulation of daf-16, the *C. elegans* ortholog of FOXO3, is known to contribute to lifespan extension in worms, it remains unclear whether this occurs through the SIRT3-UPR*^mt^* or another parallel pro-longevity pathway (Lin et al., 2001). Additionally, data showing that *daf-16* is dispensable for mtROS-induced lifespan extension argue that SIRT3/FOXO3-dependent transcriptional changes do not contribute to lifespan extension (Schmeisser et al., 2013). Additional research is warranted in this area to determine whether the transcriptional axis of the SIRT3-UPR*^mt^* is involved in lifespan extension.

##### Signaling Through Multiple Arms of the Mitochondrial Unfolded Protein Response in Aging

Alterations in ETC complexes and ROS levels appear to activate the UPR*^mt^* and affect longevity. However, these changes do not fit cleanly into a paradigm where they only activate a single arm of the UPR*^mt^*; that is, there are mitochondrial stresses that appear to simultaneously signal through multiple arms of the UPR*^mt^* to influence longevity. There is extensive evidence that manipulations of the ETC modulate longevity. Specifically, decreased expression of ETC complex subunits has been shown to extend lifespan in model organisms ranging from *C. elegans* to mice (Dillin et al., 2002; Dell’Agnello et al., 2007; Copeland et al., 2009). Importantly, it appears that the magnitude of the knockdown of ETC complex components is critical for the lifespan extending effect: severe disruptions of the ETC shorten lifespan while mild ETC disruptions extend lifespan (Rea et al., 2007). This suggests that the mitochondrial stress associated with changes in the ETC causes a mitohormetic extension of lifespan (Tapia, 2006). The link between changes in the ETC and longevity appears to be tied to ROS production and UPR*^mt^* activation (Lee et al., 2003b,2010b; Yang and Hekimi, 2010a; Rauthan et al., 2015). For example, mild knockdown of an ETC complex I component increases lifespan in *Drosophila* through a redox-dependent induction of the UPR*^mt^*, although the precise branch of the UPR*^mt^* that this effect was mediated through is not well defined (Owusu-Ansah et al., 2013). Mechanistically, data are lacking to determine specifically how ETC disruption affects lifespan, but it is likely that manipulations of the ETC alter lifespan by affecting both the SIRT3-UPR*^mt^* and ATF5-UPR*^mt^*. Because SIRT3 is a redox-sensitive protein, disruption of ETC complexes I and III, which are known to be the primary sources of ROS in the mitochondria, may activate the SIRT3-UPR*^mt^* to modulate aging (Lennicke and Cochemé, 2021). Simultaneously, ETC manipulations could create a mito-nuclear imbalance or oxidative protein damage which activates the ATF5-UPR*^mt^* to alter longevity.

It should be noted, however, that there are manipulations of the ETC that are known to activate the UPR*^mt^* without extending lifespan. For example, manipulating the expression of ETC complex II subunits does not result in long-lived worms despite the activation of the UPR*^mt^* (Ishii et al., 1998; Durieux et al., 2011; Kuang and Ebert, 2012). This may be because complex II is the only ETC complex that is solely encoded by nuclear DNA; this may suggest that the mito-nuclear imbalance, and the subsequent activation of the ATF5-UPR*^mt^*, is a critical factor in the lifespan extension (Kuang and Ebert, 2012; Houtkooper et al., 2013). It may also represent the fact that the inclusion of complex II as part of the ETC is a historical artifact; indeed, succinate dehydrogenase, as a key player in the TCA cycle, has more in common with other TCA enzymes than it does with the other ETC complexes (Munkácsy and Rea, 2014). Additionally, ETC manipulations that extend lifespan in one setting may not extend lifespan in another; for example, RNAi knockdown of a complex IV component in intestine and neurons extends lifespan in *C. elegans*, but muscle-specific knockdown of the same complex IV component does not (Durieux et al., 2011).

As discussed previously, ROS appear to modulate aging through both the ATF5-UPR*^mt^* and SIRT3-UPR*^mt^*. For example, mice with high concentrations of ROS at young ages tend to live longer than mice with lower levels of ROS; additionally, these long-lived mice show elevated levels of proteins related to the ATF5-UPR*^mt^* that persist throughout life, even after ROS levels normalize in adulthood (Latorre-Pellicer et al., 2016). Regarding the SIRT3-UPR*^mt^*, it has been shown that transient increases in ROS increase the expression of antioxidant enzymes and extend lifespan in *C. elegans*; although this study did not explicitly investigate whether the ROS-induced antioxidant gene expression was dependent upon the SIRT3-UPR*^mt^*, the similarity of the end effects leads to the speculation that transient ROS spikes may extend lifespan through the SIRT3-UPR*^mt^* (Zarse et al., 2012). Additionally, the lifespan increase of ETC mutants due to the activation of the ATF5-UPR*^mt^* is separable from the lifespan increase due to elevated levels of mtROS (Yang and Hekimi, 2010b). This suggests that increased ROS, in part, extends lifespan through pathways parallel to the ATF5-UPR*^mt^*. Again, it may be that this parallel pathway is the SIRT3-UPR*^mt^*, although this has not yet been examined. Lastly, elevated levels of mtROS increase lifespan while elevated levels of cytosolic ROS decrease lifespan providing further evidence of the potentially beneficial effects of mtROS, perhaps due to the ability of mtROS to activate the UPR*^mt^* (Schaar et al., 2015).

There are, however, numerous reports that suggest ROS can activate the UPR*^mt^* without leading to lifespan extension. In five different *C. elegans* lines with RNAi-mediated knockdown of mitochondrial proteins, the levels of oxidative stress within the animals does not correlate with lifespan (Rea et al., 2007). Additionally, treatment of long-lived *C. elegans* mitochondrial mutants with ROS-scavenging antioxidants fails to reduce lifespan despite the strong activation of the UPR*^mt^* (Durieux et al., 2011; Houtkooper et al., 2013). This suggests that another UPR*^mt^* trigger such as mito-nuclear imbalance rather than ROS explains the lifespan extension in these animals. In mice, overexpression of antioxidant genes largely has no effect on lifespan suggesting that oxidative stress and damage does not play a role in mammalian aging; however, the overexpression of antioxidants may impair the ability of a transient ROS spike to induce the UPR*^mt^* to extend lifespan (Pérez et al., 2009a). Collectively, these studies suggest that the relationship among ROS, UPR*^mt^* activation, and lifespan extension remains murky. It is likely that some combination of the timing, magnitude, and type of ROS spike is important for lifespan extension, but further research is required to determine the precise conditions that facilitate ROS- and UPR*^mt^*-dependent lifespan extension and why.

##### The Mitochondrial Unfolded Protein Response Interacts With Other Cellular Pathways in Aging

There also exist mitochondrial stress-dependent pathways that modulate aging by mechanisms not typically associated with UPR*^mt^* activation. For example, mitochondrial stress has been shown to extend lifespan, in part, through anti-viral defense mechanisms, non-canonical apoptosis signaling, cytosolic stress responses pathways, and reduced cytosolic translation (Baker et al., 2012; Delaney et al., 2013; Yee et al., 2014; Labbadia et al., 2017; Matilainen et al., 2017; Mao et al., 2020; Molenaars et al., 2020). Collectively, these data suggest that parallel cellular mechanisms play roles in the lifespan extension associated with mild mitochondrial stress and the activation of the UPR*^mt^*. Furthermore, these data may explain some discrepancies regarding the role of the UPR*^mt^* in longevity modulation.

#### Non-cell Autonomous Mitochondrial Unfolded Protein Response Signaling and Aging

Mitochondrial stress in one tissue can activate the UPR*^mt^* in tissues not experiencing mitochondrial stress, and this non-cell autonomous stress signaling has recently been shown to be involved, at least in part, in lifespan extension. To achieve this effect, it appears that two tissues are critical: neurons as the cells of stress sensing and the intestine as the tissue in which the UPR*^mt^* is activated. Durieux et al. (2011) first demonstrated that mitochondrial stress in *C. elegans* neurons non-cell autonomously activates the UPR*^mt^* in the intestine and that this signaling plays a role in lifespan extension. Other work has confirmed that this mitochondrial stress pathway from neurons to intestine promotes longevity (Tian et al., 2016; Zhu et al., 2020). Interestingly, this non-cell autonomous signaling mirrors the non-cell autonomous link between neurons and the intestine that is implicated in longevity in response to ER stress, as discussed previously (Taylor and Dillin, 2013; Frakes et al., 2020). It appears, moreover, that there are other inter-tissue communication networks that signal mitochondrial stress and influence longevity. For example, translational repression of cytochrome c in the gonad causes the non-cell autonomous activation of the UPR*^mt^* in the intestine to extend lifespan (Lan et al., 2019). However, the interconnected signaling networks that exist between reproductive and neuronal cells raises the potential that the gonad is responding to signals originating further upstream in the nervous system (Miller et al., 2020). Collectively, these data allow speculation that mitochondria in one cell type, perhaps neurons, coordinate the rate of aging for an organism through non-cell autonomous mechanisms and that the intestine may be the major tissue responsible for executing these effects. If this proves to be at least partly true, it must also be considered whether the gut plays a role in longevity not because of some intrinsic property of intestinal cells but because of the influence of the gut microbiome (Shukla et al., 2021).

#### Temporal Effects of Mitochondrial Unfolded Protein Response Activation on Aging

Across multiple studies investigating the role of UPR*^mt^* activation on longevity, a noteworthy trend has emerged: activation of the UPR*^mt^* during development appears to confer longevity benefits while initiation of the UPR*^mt^* during adulthood does not. Induction of the UPR*^mt^* during larval development is sufficient to extend lifespan and maintain the induction of the UPR*^mt^* into adulthood (Dillin et al., 2002; Rea et al., 2007; Lee et al., 2010b; Durieux et al., 2011; Borch Jensen et al., 2018; Bazopoulou et al., 2019). It appears that mitochondrial stress during development causes epigenetic changes that induce persistent UPR*^mt^*-associated gene changes into adulthood (Merkwirth et al., 2016; Tian et al., 2016; Zhu et al., 2020). However, induction of the UPR*^mt^* only during adulthood does not affect lifespan and may have detrimental effects, perhaps because of age-related epigenetic alterations that prevent access to UPR*^mt^*-induced genes (Dillin et al., 2002; Rea et al., 2007; Durieux et al., 2011; Labbadia and Morimoto, 2015; Conte et al., 2019).

### Conclusion

Collectively, data from human post-mortem tissue and animal models suggest that both the UPR*^ER^* and the UPR*^mt^* change significantly at older ages. With increasing age, both the UPR*^ER^* and the UPR*^mt^* lose adaptive signaling potency potentially leading to a detrimental state where the UPR is unable to resolve the stresses brought about by age-related cellular dysfunction (Sheng et al., 2021). It appears that modulation of the UPR*^ER^* and the UPR*^mt^* can have differing effects on age-related functional and physiological changes. For the UPR*^ER^*, manipulations can prove either beneficial or deleterious depending upon the molecular and tissue targets being investigated, and thus more research is needed to define these specific contexts. For the UPR*^mt^*, it appears that activation through the ATF5-UPR*^mt^* and SIRT3-UPR*^mt^* promotes healthy aging and lifespan extension, particularly if induced in juveniles. However, whether activation of these arms of the UPR*^mt^* is sufficient to extend lifespan remains unclear due, in part, to the technical challenges of isolating UPR*^mt^* signaling from the inciting stressors and other activated parallel stress response pathways.

## The Unfolded Protein Response in Neurodegenerative Disease

The direct physiological and functional consequences of aging are not the only drivers of age-dependent mortality; aging is also the greatest risk factor for the majority of neurodegenerative diseases (NDDs), including Alzheimer’s disease (AD), Parkinson’s disease (PD), and amyotrophic lateral sclerosis and frontotemporal dementia (ALS/FTD) (Hou et al., 2019). Two common features shared amongst the age-related NDDs are the accumulation of proteinaceous aggregates and mitochondrial dysfunction (Hou et al., 2019). Consequently, recent studies have begun to examine how stress response pathways like the UPR*^ER^* and UPR*^mt^* mitigate or aggravate cellular dysfunction in age-related NDDs. Here, we discuss the relationship between the organellar UPRs and several prominent age-related NDDs, including evidence from human post-mortem tissue of UPR activation and experimental data from model organisms demonstrating how the activation of the UPRs may alter the course of neurodegenerative pathology.

### The Endoplasmic Reticulum Unfolded Protein Response in Neurodegenerative Disease

#### The Endoplasmic Reticulum Unfolded Protein Response in Alzheimer’s Disease

Alzheimer’s disease is a NDD that primarily affects cognitive function, and represents the most common cause of dementia worldwide (Schneider et al., 2009). Histologically, AD is largely defined by the accumulation of extracellular amyloid-β (Aβ)-containing plaques and intracellular abnormal tau neurofibrillary tangles (NFTs), both of which are recognized as key, though not the only, biomarkers and possible drivers of AD pathology (Knopman et al., 2021).

A host of post-mortem studies on human brain tissue have provided evidence of UPR*^ER^* activity in AD. In the affected brain regions of AD patients, the molecular chaperone BiP is elevated in neurons at an early stage of AD pathology, suggesting that the UPR*^ER^* is activated early as a protective response in AD (Hoozemans et al., 2005). Phosphorylated IRE1α (p-IRE1α) is increased in the hippocampus of AD patients (Hoozemans et al., 2009) and has been shown to correlate with AD progression (Duran-Aniotz et al., 2017). The phosphorylated forms of PERK and eIF2α (p-PERK and p-eIF2α, respectively) are also associated with regions with AD pathology (Hoozemans et al., 2009), with p-PERK in particular concentrating in neurons with high levels of phosphorylated tau (p-tau) aggregation and markers of early NFT-induced degeneration (Unterberger et al., 2006; Hoozemans et al., 2009). Thus, a close association between IRE1α and PERK, two master regulators of the UPR*^ER^*, and the presence of p-tau appears a common feature in post-mortem AD studies. Indeed, separate studies examining brain tissue from other sporadic tauopathies, including Pick disease and progressive supranuclear palsy, have also demonstrated the correlation of both p-IRE1α and p-PERK with p-tau (Nijholt et al., 2012; Stutzbach et al., 2013). Downstream of PERK, elevated levels of ATF4, GADD34, and CHOP have also been observed in AD tissue (Yoon et al., 2012; Baleriola et al., 2014; Honjo et al., 2015). Collectively, these studies suggest that UPR*^ER^* activation represents an early event in AD pathogenesis, raising the question of whether the shift of UPR*^ER^* signaling from adaptive to chronic with disease progression may contribute to disease progression.

Studies utilizing animal models of AD have further emphasized both positive and negative roles of the IRE1α-UPR*^ER^* and PERK-UPR*^ER^* in AD pathogenesis. In *Drosophila*, *Xbp1* is upregulated following transgenic expression of either Aβ or mutant human tau and confers neuroprotection in both models (Loewen and Feany, 2010; Casas-Tinto et al., 2011). In line with this neuroprotective role, hippocampal expression of *Xbp1s* in a triple transgenic mouse model of AD (3xTg-AD) rescues structural abnormalities and memory deficits (Cissé et al., 2017). Confusingly, however, the IRE1α-UPR*^ER^* can also drive AD pathology; conditional knockout of *IRE1α* in a widely used mouse model containing five human familial AD mutations (5xFAD) restores cognitive function and reduces Aβ deposition (Duran-Aniotz et al., 2017). Likewise, *xbp-1* deficiency in nematodes reduces Aβ aggregation and toxicity (Safra et al., 2013). Thus, activity of the IRE1α-UPR*^ER^* can prove either beneficial or detrimental in AD models. This could reflect both the differences in disease model and at what stage the manipulation occurs in disease progression, which in turn may dictate whether IRE1α/XBP1 activation ameliorates or hastens neurodegenerative decline.

Elevated activity of the PERK-UPR*^ER^* is a common feature in AD patient tissue; therefore, manipulations aimed at inhibiting PERK signaling have largely been the focus in the context of AD. Suppressing eIF2α phosphorylation via the genetic deletion of *Perk* rescues deficits in spatial memory and synaptic plasticity in a double transgenic APP/PS1 mouse model of AD (Ma et al., 2013; Yang et al., 2016). Similarly, *Perk* haploinsufficiency protects against memory impairment and cholinergic neuronal loss in 5xFAD mice, concomitant with a reduction in Aβ plaque burden (Devi and Ohno, 2014). Further downstream of PERK, ATF4 may also contribute to AD pathology; knockdown of *Atf4* in axons exposed to oligomeric Aβ prevents the spreading of a degenerative signal across the mouse brain and subsequent neuronal cell loss, in part through the suppression of CHOP-mediated apoptotic signaling (Baleriola et al., 2014). Taken together, the results point to PERK signaling in AD as largely detrimental, suggesting that suppressing PERK activity may prove beneficial as an intervention in AD disease.

Little is known regarding whether the ATF6-UPR*^ER^* contributes to AD pathogenesis, though recent work has provided insight into this question. *Atf6* expression is reduced in the APP/PS1 mouse model, and overexpression of *Atf6* rescues spatial memory impairment and decreases Aβ plaque load in these mice (Du et al., 2020). Thus, the upregulation of ATF6 appears to function as a protective response in AD, though more work in independent models will be required to confirm this role.

#### The Endoplasmic Reticulum Unfolded Protein Response in Parkinson’s Disease

Parkinson’s disease is a progressive NDD most obviously associated with motor symptoms including bradykinesia, tremor, and rigidity, and is the second most prevalent age-related NDD (Poewe et al., 2017). Neuropathological hallmarks of the disease include the selective loss of dopaminergic neurons within the substantia nigra and the intracellular accumulation of proteinaceous aggregates containing α-synuclein (α-syn), referred to as Lewy bodies (Bloem et al., 2021).

In post-mortem PD tissue, increased p-PERK and p-eIF2α immunoreactivity is observed in dopaminergic neurons of the substantia nigra, and p-PERK presence correlates with neurons positive for α-syn accumulation (Hoozemans et al., 2007). In addition, α-syn aggregates accumulate within ER microsome fractions of human PD brain tissue (Colla et al., 2012). Therefore, UPR*^ER^* activation in PD appears largely in response to the deposition of α-syn aggregates, which is in agreement with *in vitro* and *in vivo* experiments demonstrating that α-syn accumulates within the ER and can activate the UPR*^ER^* via a physical interaction with BiP (Bellucci et al., 2011).

Neurotoxin-induced experimental models of PD have primarily been used to dissect the role of UPR*^ER^* signaling in PD pathophysiology. Viral overexpression of *Xbp1s* protects against dopaminergic neuron loss in a mouse model of PD induced by treatment with MPP^+^, an inhibitor of ETC complex I (Sado et al., 2009). A similar neuroprotective role for *Xbp1s* was shown in mice following treatment with 6-hydroxydopamine (6-OHDA), a generator of ROS that is acutely toxic to dopaminergic neurons (Valdés et al., 2014). Intriguingly, while reducing *Xbp1* in the adult mouse brain triggers chronic ER stress and is detrimental for neuronal survival, developmental deletion of *Xbp1* is in fact neuroprotective (Valdés et al., 2014). This developmental ablation of *Xbp1* triggers mild ER stress and subsequently drives an adaptive UPR*^ER^* program, including enhancing autophagy, that protects dopaminergic neurons from cell death (Valdés et al., 2014). A similar form of UPR*^ER^*/autophagy-mediated protection was independently observed in both fly and mouse PD models following low dosage treatment with tunicamycin (Fouillet et al., 2012), suggesting that early ER stress can prime the UPR*^ER^* for later PD-associated challenges, in part through the upregulation of autophagy. In contrast to the largely protective role of XBP1 in PD, *Ire1* expression in fly dopaminergic neurons drives JNK- and autophagy-dependent neuronal death, while its reduction ameliorates α-syn induced neurodegeneration (Yan et al., 2019). This deleterious mode of IRE1α signaling appears independent of XBP1, which therefore might represent the switch to chronic IRE1α activity in PD. Taken together, these data highlight that XBP1 expression downstream of IRE1α is neuroprotective and may represent a possible therapeutic target but that other XBP1-independent IRE1α signaling pathways have the potential to act in a deleterious fashion in PD.

The role of the other two UPR*^ER^* arms in PD pathogenesis has been less-well studied genetically. Work in murine models of PD has demonstrated that deletion of the PERK downstream apoptotic regulator *Chop* protects against 6-OHDA-induced dopaminergic neuron death (Silva et al., 2005), suggesting that the pro-apoptotic arm of PERK signaling is induced and is detrimental for dopaminergic neuron survival. Conversely, the role of ATF6 appears neuroprotective in the context of PD. Following MPP^+^ treatment, Atf6 promotes astrocytic activation and the upregulation of chaperones and ERAD genes to prevent dopaminergic neurodegeneration (Egawa et al., 2011; Hashida et al., 2012). Taken together with the previously discussed protective function of XBP1 in PD, signaling through both the PERK-UPR*^ER^* and the ATF6-UPR*^ER^* appear to augment neuroprotective programs in PD models. This conclusion is supported by a recent study demonstrating that viral-mediated delivery of an XBP1/ATF6 fusion protein promotes degradation of α-syn aggregates *in vivo* and protects dopaminergic neurons following 6-OHDA treatment (Vidal et al., 2021). Therefore, the promotion of UPR*^ER^* genes downstream of XBP1 and ATF6, including chaperones and degradative pathways, displays neuroprotective potential in PD pathogenesis.

#### The Endoplasmic Reticulum Unfolded Protein Response in Amyotrophic Lateral Sclerosis and Frontotemporal Dementia

Amyotrophic lateral sclerosis is a NDD characterized cellularly by motor neuron death and clinically by muscle weakness and atrophy leading to paralysis and death typically within 2–5 years of disease onset (Harris, 2014). Additionally, approximately 40% of ALS patients present with cognitive and behavioral impairments with half of these patients being diagnosed with behavioral variant FTD (Masrori and Van Damme, 2020). Indeed, ALS and FTD display significant clinical and genetic overlap and are now considered to be on a spectrum; lesions in several genes, including *TAR-DNA binding protein 43* (*TDP-43*) and *chromosome 9 open reading frame 72* (*c9ORF72*), can cause ALS, FTD, or both (Abramzon et al., 2020). The majority of ALS/FTD subtypes can be characterized by the presence of cytoplasmic proteinaceous aggregates positive for TDP-43 (Sreedharan et al., 2008). However, in other subtypes different protein species can be present such as misfolded superoxide dismutase (SOD1) or dipeptide repeat proteins (DPRs) produced from a mutant form of *c9ORF72*, the highest frequency familial ALS/FTD causing lesion (Hardiman et al., 2017).

The activation of UPR*^ER^* chaperones appears to be a common feature in ALS patient samples. Both BiP and PDI have been observed in ALS spinal cord tissue (Ilieva et al., 2007; Sasaki, 2010; Montibeller et al., 2020), as have UPR*^ER^* transcription factors XBP1 (Hetz et al., 2009) and CHOP (Ito et al., 2009). Furthermore, BiP and PDI are represented among a cluster of chaperones upregulated in ALS peripheral blood mononuclear cells (Nardo et al., 2011). Finally, a recent transcriptome analysis of *c9ORF72* ALS patient tissue identified widespread alterations to genes involved in the UPR*^ER^* in both cerebellum and cortex samples (Prudencio et al., 2015). UPR*^ER^* activation is also associated with FTD; p-IRE1α, p-PERK, and p-eIF2α are elevated in *c9ORF72* FTD patient samples, observed in close association with DPR inclusions (Gami-Patel et al., 2021). Therefore, the presence of markers of UPR*^ER^* activation appear across the ALS/FTD continuum, suggesting that UPR*^ER^* activity may represent a common pathological feature amongst the range of lesions known to cause these diseases.

Experimental models of ALS/FTD have been used to investigate the contribution of upregulated UPR*^ER^* components to disease pathology. Knockdown of *IRE1α* or *Xbp1* reduces mutant SOD1 aggregation in motor neurons both *in vitro* and in mutant SOD1 mice, subsequently extending lifespan and reducing apoptotic cell death (Hetz et al., 2009). Intriguingly, *Xbp1* deficiency impairs ERAD function but upregulates autophagy, suggesting that compensatory degradative pathways are activated in this paradigm (Hetz et al., 2009). Initial studies using mutant SOD1 mice demonstrated that the PERK-UPR*^ER^* acts protectively in ALS pathogenesis (Wang et al., 2011, 2014a), though a more recent study using several strains of mutant SOD1 mice demonstrated that manipulating the PERK pathway had no effect on disease onset or progression, which could reflect relative differences in mutant SOD1 expression levels between mice strains (Dzhashiashvili et al., 2019). However, downstream of PERK, deleting *Atf4* in mutant SOD1 mice reduces developmental viability but ameliorates disease phenotypes, possibly via the reduction of apoptotic effector CHOP (Matus et al., 2013). Thus, the true contribution of the PERK-UPR*^ER^* to mutant SOD1 forms of ALS/FTD remains up for debate, whilst its participation in other forms of ALS/FTD pathology is largely unexplored.

### The Mitochondrial Unfolded Protein Response in Neurodegenerative Disease

#### The Mitochondrial Unfolded Protein Response in Alzheimer’s Disease

Data from both animal models and humans show that the UPR*^mt^* is intimately involved in AD pathobiology. Genes involved in the UPR*^mt^*, such as *YMEL1L*, which encodes a mitochondrial AAA + protease and *HSP60*, which encodes a chaperone, are upregulated in post-mortem brain tissue obtained from patients with AD (Beck et al., 2016; Sorrentino et al., 2017). Upregulation of UPR*^mt^*-related genes is also recapitulated in the nervous systems of animal models of AD (Sorrentino et al., 2017; Shen et al., 2020). Based on data showing that Aβ accumulation co-occurs with mitochondrial import impairments, Sorrentino et al. (2017) argue that Aβ may impair mitochondrial import efficiency to trigger the UPR*^mt^*. Additionally, the finding that resveratrol, an activator of sirtuins, reduces Aβ toxicity in a ubl-5-dependent fashion suggests that Aβ may activate both the ATF5-UPR*^mt^* and SIRT3-UPR*^mt^* (Regitz et al., 2016). Regardless of the mechanism by which the UPR*^mt^* is activated in AD, it is clear from multiple independent studies that the activation is largely beneficial. Brief exposure of human neuroblastoma-derived SH-SY5Y cells to Aβ fragments causes the accumulation of the mitochondrial proteases LONP1, HTRA2, and CLPP and as well as the mitochondrial chaperone HSP60, indicating that the UPR*^mt^* has been activated (Shen et al., 2020). This induction of the UPR*^mt^* prevents Aβ-induced cellular toxicity which suggests that acute UPR*^mt^* activation may represent a beneficial compensatory cellular response (Shen et al., 2020). Additionally, the overexpression of *atfs-1* in a *C. elegans* model of AD further induces the UPR*^mt^* and improves healthspan suggesting that the induction of the UPR*^mt^* offsets some of the pathological effects of Aβ aggregation (Sorrentino et al., 2017). Lastly, inhibition of the UPR*^mt^* worsens cellular features commonly associated with AD such as an increased Aβ42:Aβ40 ratio and increased phosphorylated tau in an AD-like human cerebral organoid model (Pérez et al., 2020).

#### The Mitochondrial Unfolded Protein Response in Parkinson’s Disease

Mitochondrial dysfunction is a key player in the pathophysiology of PD, and many genetic and toxic causes of PD are also known to activate the UPR*^mt^* (Pellegrino and Haynes, 2015; Bloem et al., 2021). To our knowledge, no one has yet completed a comprehensive study of post-mortem brain tissue from PD patients examining multiple markers of UPR*^mt^* activation. However, transcript and protein levels of UPR*^mt^*-related proteases and chaperones are increased in human cell lines exposed to the PD-causing toxin MPP^+^ suggesting that the UPR*^mt^* may indeed be activated in PD (Cai et al., 2020). Bolstering evidence that the UPR*^mt^* plays a role in PD in humans is the finding that some PD patients have mutations in *HTRA2*, a key player in the ERα-UPR*^mt^* (Strauss et al., 2005; Unal Gulsuner et al., 2014). In animal and cell models of PD, the activation of the UPR*^mt^* has been shown to exert both protective and damaging effects. In MPP^+^-treated SH-SY5Y cells, further activation of the UPR*^mt^* reduces cell death (Hu et al., 2021). Additionally, in genetic worm models of PD, inactivation of the UPR*^mt^* decreases lifespan and worsens dopaminergic neuron loss (Cooper et al., 2017). Furthermore, overexpression of the UPR*^mt^* component *CLPP*, which encodes a mitochondrial matrix protease, in induced pluripotent stem cell-derived neurons from PD patients reduces pathogenic α-syn phosphorylation, reduces mitochondrial oxidative stress, and restores neuron morphology (Hu et al., 2019). In contrast, Martinez et al. (2017) found that chronic over-activation of the UPR*^mt^* is detrimental for lifespan and healthspan in *C. elegans* overexpressing a mutant form of α-syn. It is likely that this chronic UPR*^mt^* hyperactivation exerts detrimental cellular effects in concert with aggregates of α-syn. Mechanistically, it appears that aggregation-prone α-syn accumulates within mitochondria to activate the UPR*^mt^* directly (Hu et al., 2019). Overall, it appears that the UPR*^mt^* is beneficial under many circumstances, save those where the UPR*^mt^* is chronically over-activated.

#### The Mitochondrial Unfolded Protein Response in Amyotrophic Lateral Sclerosis and Frontotemporal Dementia

Mounting evidence suggests that the UPR*^mt^* is activated in the context of ALS/FTD-related mutant TDP-43, SOD1, CHCHD10, and FUS; however, it remains unclear what role this UPR*^mt^* activation plays in the ALS/FTD pathobiology (Riar et al., 2017; Deng et al., 2018; Anderson et al., 2019; Pharaoh et al., 2019; Wang et al., 2019; Straub et al., 2021). Mutant TDP-43 induces the UPR*^mt^*, likely through inhibition of ETC complex I leading to reduced mitochondrial ATP synthesis, in both human cell lines and transgenic *Drosophila* models of ALS/FTD (Wang et al., 2019). In these models, downregulation of the mitochondrial protease LONP1 increased TDP-43 protein levels and exacerbated TDP-43-induced mitochondrial dysfunction and neurodegeneration suggesting that the UPR*^mt^* acts to counter the detrimental effects of TDP-43 (Wang et al., 2019). In transgenic mice expressing mutant human *SOD1*, the ATF5-UPR*^mt^* appears to be activated in the early stages of ALS disease in mouse spinal cord (Pharaoh et al., 2019). Additionally, mutant SOD1, which is known to accumulate in the mitochondrial IMS to cause mitochondrial dysfunction, appears to upregulate ERα-UPR*^mt^* effectors such as the IMS protease *HTRA2* (Riar et al., 2017). Mutations in *CHCHD10* cause mitochondrial dysfunction, impaired mitochondrial proteostasis, and the activation of the UPR*^mt^* (Bannwarth et al., 2014; Anderson et al., 2019; Straub et al., 2021). The loss of CHCHD10 appears to activate both the ATF5-UPR*^mt^* and SIRT3-UPR*^mt^* as evidenced by enhanced activation of the transcription factors ATF4, ATF5, and FOXO3 (Straub et al., 2021). Lastly, in cell lines and flies, the overexpression of the *FUS* leads to disruption of the ATP synthase complex and activation of the UPR*^mt^*, both of which contribute to the FUS-induced neurodegeneration (Deng et al., 2018). These disparate findings potentially suggest that different ALS/FTD-related mutations have differing effects on the UPR*^mt^*, although it cannot be ruled out that differences in disease models account for the contradictory effects of UPR*^mt^* activation on cellular survival.

### Conclusion

The inability of cells and tissues to maintain proteostasis, including mitochondrial proteostasis, appears to be central to the pathophysiology of many age-related NDDs (Hipp et al., 2014; Hou et al., 2019). Thus, it is no surprise that evidence of UPR*^ER^* and UPR*^mt^* activation is a common feature in patient post-mortem nervous system tissue. Whether this activation represents protective or detrimental signaling is complex. Activation of the UPR*^ER^* by virally delivered XBP1 or XBP1/ATF6 fusion protein appears to be protective across multiple NDD contexts (Sado et al., 2009; Zuleta et al., 2012; Cissé et al., 2017; Vidal et al., 2021). However, it is also clear that dysregulated UPR*^ER^* signaling can drive pathogenesis under certain disease states, and so future work aimed at better defining both protective and detrimental UPR*^ER^* signatures in disease models will be critical moving forward to allow targeting of therapies to the appropriate disease populations. Disease stage and context also appear to define the contribution of the UPR*^mt^* to disease onset and progression. The currently available data paint a picture where the UPR*^mt^* is acutely activated early in the course of age-related NDD to preserve cellular health. At this stage, it is likely that moderate levels of proteostasis impairment trigger the UPR*^mt^* to preserve mitochondrial and cellular integrity and health. This view is bolstered by data suggesting that inducing the accumulation of proteins within the mitochondria does not lead to cell death but does activate the UPR*^mt^* (Poveda-Huertes et al., 2020). However, data also suggest that continued activation or hyper-activation of the UPR*^mt^*, likely in response to chronic or severe proteotoxic stress that occurs with the progression of age-related NDD, can elicit detrimental effects (Kenny and Germain, 2017; Martinez et al., 2017; Hou et al., 2019). Thus, the relationship between the organellar UPRs and NDD is complex, and more work is needed to understand these relationships more completely.

## Therapeutics and the Unfolded Protein Response

Modification of UPR*^ER^* and UPR*^mt^* signaling has been shown to affect aging and age-related NDDs. Pharmacological agents targeting the UPR*^ER^* and UPR*^mt^* may, therefore, hold promise as therapeutics (see Table 2). Here, we review several potential therapeutic strategies seeking to alter UPR*^ER^* and UPR*^mt^* signaling to elicit beneficial effects; we also discuss the next steps needed to translate these pre-clinical findings into clinical therapeutics. For a more comprehensive review of therapeutic strategies targeting UPR function, including chemical chaperones, we refer readers to a recent review (Marciniak et al., 2021).

**TABLE 2:** Therapeutics targeting either the UPR*^ER^* or the UPR*^mt^*, their mechanisms of action, and their effects in various model systems.

Therapeutic	UPR target and mechanism of action	Effect(s)	Model(s)	References
Salubrinal	**↑ PERK-UPR*^ER^*** Inhibits eIF2α dephosphorylation	Rescues motor impairment, reduces mortality	Mutant SOD1-expressing ALS mice	Saxena et al., 2009
		Confers neuroprotection	Aβ-expressing human neuronal cells	Lee et al., 2010a
		Exacerbates neuronal loss, reduces lifespan	Prion-infected mice	Moreno et al., 2012
		Decreases α-syn accumulation, rescues motor impairment	α-syn-expressing PD mice and rats	Colla et al., 2012
		Confers neuroprotection	Rotenone-induced PD human neuronal cells	Wu et al., 2014
Guanabenz	**↑ PERK-UPR*^ER^*** Inhibits eIF2α dephosphorylation	Promotes clearance of abnormal prions (PrP*^Sc^*)	Yeast and prion-infected mouse Schwann cells	Tribouillard-Tanvier et al., 2008
		Delays disease progression, reduces mortality	Mutant SOD1-expressing ALS mice	Jiang et al., 2014; Wang et al., 2014b
		Accelerates disease progression	Mutant SOD1-expressing ALS mice	Vieira et al., 2015
		Ameliorates pathology	Mutant SOD1-expressing ALS mice	Das et al., 2015
		Extends lifespan	Prion-infected mice	Thapa et al., 2020
GSK2606414	**↓ PERK-UPR*^ER^*** Inhibits PERK activation	Confers neuroprotection	Prion-infected mice	Moreno et al., 2013
		Lowers p-tau, confers neuroprotection	Mutant tau-expressing FTD mice	Radford et al., 2015
		Confers neuroprotection but also causes pancreatic toxicity	Neurotoxin-induced PD mice	Mercado et al., 2018
ISRIB	**↓ PERK-UPR*^ER^*** Reverses phosphorylation of eIF2α	Confers neuroprotection	Prion-infected mice	Halliday et al., 2015
		Confers neuroprotection	Mutant SOD1-expressing ALS rat neuronal cells	Bugallo et al., 2020
		Rescues memory impairment	Wild-type mice	Krukowski et al., 2020
Trazodone	**↓ PERK-UPR*^ER^*** Inhibits p-eIF2α signaling	Confers neuroprotection	Prion-infected mice, mutant tau-expressing FTD mice	Halliday et al., 2017
IXA1/IXA4/IXA6	**↑ IRE1α-UPR*^ER^*** Specifically activates IRE1-dependent XBP1 signaling	Reduces Aβ levels (IXA4) and reduces APP secretion (IXA4/6)	Mutant APP-expressing AD hamster cells	Grandjean et al., 2020
Doxycycline	**↑ ATF5-UPR*^mt^*** Induces mito-nuclear imbalance	Extends lifespan	Wild-type *C. elegans*	Houtkooper et al., 2013
		Confers neuroprotection, reduces mortality	Aβ-expressing *C. elegans*	Sorrentino et al., 2017
Chloramphenicol	**↑ ATF5-UPR*^mt^*** Induces mito-nuclear imbalance	Extends lifespan	Wild-type *C. elegans*	Houtkooper et al., 2013
Nicotinamide riboside	**↑ ATF5-UPR*^mt^*** Induces mito-nuclear imbalance, and **↑ SIRT3-UPR*^mt^*** Increases [NAD^+^]	Extends lifespan	Wild-type yeast	Belenky et al., 2007
		Extends lifespan	Wild-type *C. elegans*	Mouchiroud et al., 2013
		Confers neuroprotection, reduces mortality	Aβ-expressing *C. elegans*	Sorrentino et al., 2017
		Reduces Ab toxicity, rescues memory impairment	Mutant APP- and PSEN1-expressing AD mice	Sorrentino et al., 2017
Nicotinamide mononucleotide	**↑ ATF5-UPR*^mt^*** Likely induces mito-nuclear imbalance, and **↑ SIRT3-UPR*^mt^*** Increases [NAD^+^]	Extends lifespan	Wild-type *C. elegans*	Mouchiroud et al., 2013
		Confers neuroprotection	Mutant PINK1-expressing PD *D. melanogaster*	Lehmann et al., 2017
Olaparib (also called AZD2281)	**↑ ATF5-UPR*^mt^*** Induces mito-nuclear imbalance, and **↑ SIRT3-UPR*^mt^*** Increases [NAD^+^]	Extends lifespan	Wild-type *C. elegans*	Mouchiroud et al., 2013
		Confers neuroprotection, reduces mortality	Aβ-expressing *C. elegans*	Sorrentino et al., 2017
Resveratrol	**↑ ATF5-UPR*^mt^*** Induces mito-nuclear imbalance, and **↑ SIRT3-UPR*^mt^*** Activates sirtuins	Extends lifespan	Wild-type *C. elegans*	Houtkooper et al., 2013
		Confers neuroprotection	Aβ-expressing *C. elegans*	Regitz et al., 2016

### Therapeutics Targeting the Endoplasmic Reticulum Unfolded Protein Response

#### Therapeutics Targeting the Inositol-Requiring Enzyme 1α-Endoplasmic Reticulum Unfolded Protein Response

Pharmacological targeting of the IRE1α-UPR*^ER^* has received little attention in the context of aging and NDD. However, a recent study screening for compounds that activate *XBP1* splicing through IRE1α identified three promising options. IXA1, IXA4, and IXA6 selectively activate XBP1 signaling without affecting other functions of IRE1α, and IXA4 and IXA6 reduce levels of Aβ by improving proteostasis (Grandjean et al., 2020). Thus, selective pharmacological targeting of XBP1 may offer a therapeutic strategy to activate protective UPR*^ER^* signaling and further testing of these strategies in NDD models is warranted.

#### Therapeutics Targeting the Protein Kinase RNA-Like Endoplasmic Reticulum Kinase-Endoplasmic Reticulum Unfolded Protein Response

In contrast to the IRE1α-UPR*^ER^*, pharmacological modulation of the PERK-UPR*^ER^* signaling pathway has been investigated extensively. Enhancing PERK-eIF2α signaling through the inhibition of eIF2α dephosphorylation has provided mixed results, with evidence that it can either protect (Saxena et al., 2009; Vaccaro et al., 2013; Jiang et al., 2014; Wang et al., 2014b; Das et al., 2015) or exacerbate (Moreno et al., 2012; Vieira et al., 2015) NDDs pathology in different animal models. Pharmacological inhibition of PERK kinase activity has also demonstrated neuroprotection in mice (Moreno et al., 2013; Radford et al., 2015; Mercado et al., 2018), but also can cause pancreatic toxicity as a secondary effect (Mercado et al., 2018), highlighting the need to further characterize the functional consequences of PERK kinase inhibition to organismal health. Indeed, partial inhibition of signaling downstream of PERK has proven effective at ameliorating NDD pathology without causing secondary toxicity (Halliday et al., 2015, 2017; Bugallo et al., 2020), and has also been demonstrated to ameliorate natural age-related cognitive decline in old mice (Krukowski et al., 2020), suggesting that fine-tuning PERK signaling as opposed to total inhibition may prove a better therapeutic strategy to target aging and age-related NDD.

#### Therapeutics Targeting the Activating Transcription Factor 6-Endoplasmic Reticulum Unfolded Protein Response

Similar to the IRE1α-UPR*^ER^*, pharmacological strategies targeting ATF6 in aging and neurodegeneration have not yet been thoroughly explored. A recent screen to identify small regulators of ER proteostasis identified a number of regulators of ATF6-dependent transcriptional pathways (Plate et al., 2016). One compound in particular, named AA147, demonstrated effectiveness in reducing the secretion and aggregation of several proteotoxic species, including amyloidogenic immunoglobulin light chain (ALLC) (Plate et al., 2016). Moving forward, it will be of interest to determine whether these compounds are also capable of reducing protein inclusions characteristic of many NDDs.

### Therapeutics Targeting the Mitochondrial Unfolded Protein Response

#### Therapeutics Targeting the Activating Transcription Factor 6-Mitochondrial Unfolded Protein Response

Two therapeutic strategies appear capable of activating the ATF5-UPR*^mt^* to avert the deleterious effects of aging and neurodegeneration. First, antibiotics that inhibit mitochondrial protein synthesis, namely doxycycline and chloramphenicol, have been shown to upregulate the expression of ATF5-dependent UPR*^mt^* genes and extend lifespan in *C. elegans* (Houtkooper et al., 2013). Furthermore, repression of protein synthesis can also protect against Aβ and α-syn aggregation and neurotoxicity by inducing the expression of UPR*^mt^*-linked genes (Sorrentino et al., 2017; Dastidar et al., 2020). Second, drugs that increase the concentration of NAD^+^, or mimic this effect, appear to activate the ATF5-UPR*^mt^* and SIRT3-UPR*^mt^* to extend lifespan and protect against NDD-related cellular and organismal decline in various model systems (Belenky et al., 2007; Houtkooper et al., 2013; Mouchiroud et al., 2013; Regitz et al., 2016; Lehmann et al., 2017; Sorrentino et al., 2017).

#### Therapeutics Targeting the Estrogen Receptor Alpha-Mitochondrial Unfolded Protein Response

Currently, there exist no drugs targeting the ERα-UPR*^mt^* that have been specifically investigated in the context of aging or neurodegeneration. As current data suggest that the ERα-UPR*^mt^* does not play a role in modulating aging nor neurodegeneration, more research is needed in this area to assess whether pharmacological targeting of this pathway is a worthwhile endeavor.

#### Therapeutics Targeting the SIRT3-Mitochondrial Unfolded Protein Response

As discussed previously, many NAD^+^ modulators not only activate the ATF5-UPR*^mt^* but also the SIRT3-UPR*^mt^*. As drugs that increase the cellular NAD^+^ concentration appear to extend lifespan and protect against neurodegeneration in model systems, future research investigating whether activation of the SIRT3-UPR*^mt^* independently of the ATF5-UPR*^mt^* achieves these same effects could aid in the design of more targeted therapeutics with the potential to increase resistance to the ill-effects of aging and NDD.

### Conclusion

Taken together, current data suggest that targeting either the UPR*^ER^* or UPR*^mt^* may hold promise as a therapeutic strategy to combat the ill-effects of aging and NDDs. However, many issues remain to be solved before therapeutic strategies targeting the UPR*^ER^* and UPR*^mt^* can be translated to humans. First, it remains a key question how best to harness beneficial UPR signaling without inducing the pathological effects of chronic hyperactivity, though recent studies of the UPR*^ER^* support the idea that fine-tuning UPR activity may provide a better template than directly targeting the master regulators. Second, it remains to be seen whether different neuronal subtypes respond similarly to modulators of the UPRs; activation of either of the organellar UPRs may be beneficial in some neurons but harmful in others. Third, as both the UPR*^ER^* and UPR*^mt^* are composed of multiple, parallel signaling pathways, more research is needed to assess how individual pathways may compensate for drug-induced changes in another. Fourth, activation of the UPR*^ER^* or UPR*^mt^* appears to be beneficial in some contexts and detrimental in others. Therefore, UPR-modifying therapies will rely upon the identification of biomarkers that can differentiate beneficial from detrimental circumstances. Lastly, numerous reports have suggested that there are non-cell autonomous effects of activating the UPR in one tissue; understanding how the activation of the UPR in multiple tissues simultaneously affects organismal health will be important when considering if UPR modulators represent an avenue to stave off the detrimental effects of aging and NDD.

## Conclusion

The decline of proteostasis and mitochondrial dysfunction are both closely tied to the deterioration of organismal health, and it is clear from the data collected thus far that the UPR*^ER^* and UPR*^mt^* both have complex relationships with aging and age-related NDDs. These relationships share common features as both UPRs experience a functional decline with age, while evidence of their activation is frequently observed in post-mortem NDD patient tissue. Additionally, genetic studies in model organisms demonstrate that manipulations of either UPR can influence aging and neurodegeneration, though these changes remain complex and context dependent. Activating specific components of either mechanism can prove beneficial in ameliorating disease phenotypes and extending lifespan under some circumstances, though sustained activation results in dysregulated signaling and can instead exacerbate pathology. Thus, how to best harness beneficial UPR functionality, while avoiding the consequences of sustained activity, remains a critical question when considering the therapeutic potential of UPR modulation for aging and age-related NDDs.

## Author Contributions

APKW and AWS conducted the literature search and wrote the first draft of the manuscript. APKW designed the figures. All authors contributed to conceptualizing, reviewing, and editing the manuscript, approved the submission of the manuscript.

## Conflict of Interest

The authors declare that the research was conducted in the absence of any commercial or financial relationships that could be construed as a potential conflict of interest.

## Publisher’s Note

All claims expressed in this article are solely those of the authors and do not necessarily represent those of their affiliated organizations, or those of the publisher, the editors and the reviewers. Any product that may be evaluated in this article, or claim that may be made by its manufacturer, is not guaranteed or endorsed by the publisher.

## Abbreviations

α-syn: alpha synuclein
A β: amyloid beta
AD: Alzheimer’s disease
ALS/FTD: amyotrophic lateral sclerosis/frontotemporal dementia
ATF4: activating transcription factor 4
ATF5: activating transcription factor 5
ATF6: activating transcription factor 6
Bcl2: B cell lymphoma 2
BiP: binding immunoglobulin protein
cbp-1: CREB binding protein 1
CHOP: C/EBP homologous protein
DPR: dipeptide repeat
DR: dietary restriction
eIF2 α: elongation initiation factor alpha
ER: endoplasmic reticulum
ER α: estrogen receptor alpha
ERAD: ER-associated degradation
ETC: electron transport chain
IMS: inner membrane space
IRE1 α: inositol-requiring enzyme 1 alpha
ISC: intestinal stem cell
MIM: mitochondrial inner membrane
miRNA: microRNA
MOM: mitochondrial outer membrane
mPTP: mitochondrial permeability transition pore
mtROS: mitochondrial reactive oxygen species
NDD: neurodegenerative disease
PD: Parkinson’s disease
PDI: protein disulfide isomerase
PERK: protein kinase RNA-like endoplasmic reticulum kinase
Pomc: proopiomelanocortin
PP1: protein phosphatase 1
RIDD: regulated IRE1-dependent decay
ROS: reactive oxygen species
S1P: site-1 protease
S2P: site-2 protease
SCV: small clear vesicle
SOD1: superoxide dismutase
UPR: unfolded protein response
UPR*^ER^*: endoplasmic reticulum unfolded protein response
UPR*^mt^*: mitochondrial unfolded protein response
XBP1: X-box binding protein 1
XBP1s: X-box binding protein 1, spliced.
